# Lipidome determinants of maximal lifespan in mammals

**DOI:** 10.1038/s41598-017-00037-7

**Published:** 2017-01-31

**Authors:** Katarzyna Bozek, Ekaterina E. Khrameeva, Jane Reznick, Damir Omerbašić, Nigel C. Bennett, Gary R. Lewin, Jorge Azpurua, Vera Gorbunova, Andrei Seluanov, Pierrick Regnard, Fanelie Wanert, Julia Marchal, Fabien Pifferi, Fabienne Aujard, Zhen Liu, Peng Shi, Svante Pääbo, Florian Schroeder, Lothar Willmitzer, Patrick Giavalisco, Philipp Khaitovich

**Affiliations:** 1 CAS Key Laboratory of Computational Biology, CAS-MPG Partner Institute for Computational Biology, 320 Yue Yang Road, Shanghai, 200031 China; 2 Max Planck Institute for Evolutionary Anthropology, Deutscher Platz 6, 04103 Leipzig, Germany; 3 Skoltech Center for Computational and Systems Biology, Skolkovo Institute for Science and Technology, Novaya st. 100, Skolkovo, 143025 Russia; 4 Institute for Information Transmission Problems, Russian Academy of Sciences, Bolshoy Karetny per. 19/1, Moscow, 127051 Russia; 5 Max-Delbrück Center for Molecular Medicine, Department of Zoology and Entomology, University of Pretoria, Pretoria, Republic of South Africa South Africa; 6 Max-Delbrück Center for Molecular Medicine, Robert-Rössle-Str. 10, 13092 Berlin, Germany; 7 Department of Zoology and Entomology, University of Pretoria, Pretoria, Republic of South Africa South Africa; 8 University of Rochester, 434 Hutchison Hall, Rochester, NY 14627-0211 USA; 9 SILABE, Primatology Center, University Louis Pasteur, Strasbourg, 4 Rue Blaise Pascal, 67070 France; 10 UMR 7179/CNRS-MNHN, 1 avenue du Petit Chateu, 91800 Brunoy, France; 11 State Key Laboratory of Genetic Resources and Evolution, Kunming Institute of Zoology, Chinese Academy of Sciences, 32 Jiaochang Dong Lu, Kunming, Yunnan 650223 China; 12 Max Planck Institute for Molecular Plant Physiology, Am Mühlenberg 1, 14476 Potsdam, Germany; 13 Present Address: Okinawa Institute of Science and Technology Graduate University, 1919-1 Tancha, Onna-son, Kunigami-gun, Okinawa 904-0495 Japan

**Keywords:** Molecular evolution, Computational science

## Abstract

Maximal lifespan of mammalian species, even if closely related, may differ more than 10-fold, however the nature of the mechanisms that determine this variability is unresolved. Here, we assess the relationship between maximal lifespan duration and concentrations of more than 20,000 lipid compounds, measured in 669 tissue samples from 6 tissues of 35 species representing three mammalian clades: primates, rodents and bats. We identify lipids associated with species’ longevity across the three clades, uncoupled from other parameters, such as basal metabolic rate, body size, or body temperature. These lipids clustered in specific lipid classes and pathways, and enzymes linked to them display signatures of greater stabilizing selection in long-living species, and cluster in functional groups related to signaling and protein-modification processes. These findings point towards the existence of defined molecular mechanisms underlying variation in maximal lifespan among mammals.

## Introduction

In contrast to average life expectancy that may change depending on living conditions, maximal lifespan (MLS) is a stable characteristic of a species. For instance, human average life expectancy has increased substantially over the past centuries, but our MLS remained constant at approximately 120 years^1^. At the same time, MLS varies greatly among species. In mammals it ranges from 3–4 years in small rodents to as long as 150–200 years in bowhead whales^2^. More remarkably, MLS evolves rapidly, resulting in markedly different lifespans and, consequently, different time of aging onset, even among closely related species. For instance, humans and macaques diverged only around 30 million years ago (MYA), yet over this time their MLS has diverged as much as three-fold^3^.

Despite remarkable evolutionary plasticity of MLS, the existence and nature of the molecular mechanisms controlling this variation remains unclear. Most studies so far have focused on mechanisms of lifespan plasticity within species, resulting in identification of longevity-related pathways, such as insulin signaling pathways and targets of rapomycin pathway, shared across a wide range of animal species: from worms to mice^4–8^. Genetic, dietary and pharmacological manipulation targeting these pathways resulted in more than 10-fold lifespan extension in short-living nematode worms^9^, but only approximately 40% (1.4-fold) lifespan extension in short-living mammals such as laboratory mice^10^. At the same time, natural variation in MLS among mammalian species exceeds 50-fold^2^.

In this study we searched for a link between MLS and another marker of species’ physiology – concentrations of hydrophobic metabolic compounds (henceforth referred to as “lipids” for simplicity). Recent scan of gene expression variation among 33 mammalian species with MLS differences of over 30-fold has shown that expression variation of 11–18% of analyzed 19,643 genes could be associated with MLS variation^11^. There is however a stronger evidence of genetic changes in genes controlling lipid metabolism to play a role in human longevity^11–16^, as well as in MLS differences among species^17^, and changes in lipid saturation levels^18–25^, pointing to lipids as a good potential target for investigation of molecular mechanisms underlying differences in MLS among mammalian species.

## Results

### Data samples

**Figure 1:**
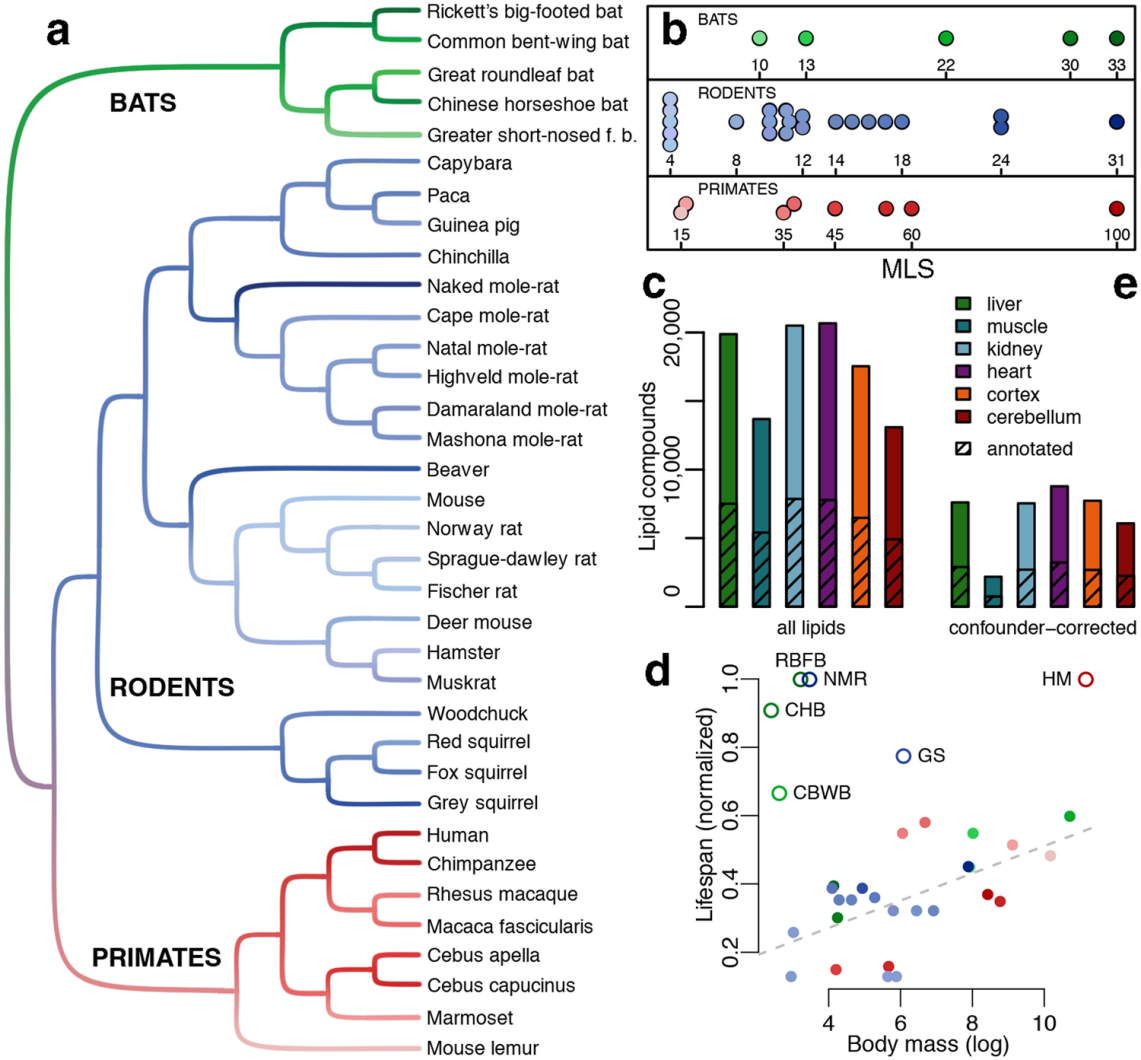
Dataset. (**a**) Phylogenetic tree of the 35 species used in this study. Colors show the clade identity and the MLS of species, with darker shades representing longer MLS. (**b**) Species’ MLS distribution in years. Each dot represents a species. Colors are as in panel a. (**c**) Number of lipid compounds measured in each tissue. (**d**) Relationship between species’ MLS and body mass. The colors are as in panel a. Open circles indicate species deviating from the linear relationship between MLS and body mass. The dashed line shows linear model fit to the remaining species (F-test, p < 0.05). MLS was normalized to the maximal MLS value within each clade. NMR – naked mole-rat, HM – human, GS – grey squirrel, CBWB – common bent-winged bat, RBFB – rickett’s big-footed bat, CHB – chinese horseshoe bat. (**e**) Number of lipid compounds of each tissue after removal of the compounds related to the confounding factors.

To investigate the direct connection between lipid metabolic processes and MLS, we measured lipid concentrations in liver, muscle, kidney, heart, brain cortex and cerebellum samples in a total of 669 individuals of 35 mammalian species representing three phylogenetic clades: rodents, primates, and bats (Fig. 1a, Tables S1 and S2). Within clades, MLS varied between 4 and 31 years in rodents, 15 and 100 years in primates, and 10 and 33 years in bats (Fig. 1b)^26^. As long-living species we consider those with MLS exceeding 0.9 of the highest MLS in each clade. Based on this cutoff human, naked mole-rat and two bat species (chinese horseshoe bat and rickett’s big-footed bat) are regarded as long-living throughout this study.

Using liquid chromatography coupled with high-precision mass spectrometry in positive and negative ionization modes, we quantified between 13,000 and 21,000 hydrophobic compounds with molecular weights below 3,000 Daltons, detected in at least 50% of individuals in one tissue of one species. Among them, between 5,000 and 8,000 (38%) were annotated using computational matching to the lipid compound database (Fig. 1c, Table S3)^27^.

Several factors, such as species’ basal metabolic rate (BMR), body mass and temperature, diet, and ability to hibernate, have been shown to explain some, but not all, variability in species’ lifespan. To discriminate the effect of these factors from the effect of lipid concentrations on MLS, our dataset included species where MLS was uncoupled from one or more of these factors (Figs 1d, S1). All lipids that were significantly related to these factors, as well as to individuals’ age and sex (Figure S2, Table S4), were removed from further analyses (Fig. 1e).

### Predictive models of MLS

To search for combinations of lipidome features that might be predictive of species’ MLS, we constructed models distinguishing long-living species in all three clades from other species, based on the concentrations of lipid compounds in each tissue. The models were based on logistic regression with elastic net penalty – an approach which by applying a penalty measure on the number of lipids selected for the model during the training process selects only their limited number. This way we use this predictive model as a tool to both assess how accurately can we estimate long lifespan based on lipidome, as well as to investigate which lipid compounds are essential for this estimation and therefore potentially play a role in the species’ exceptionally long lifespans. In each tissue, we performed 10 × 10 cross-validation to determine optimal model parameters and to assess predictive accuracy. Since other classification methods, as well as linear models, resulted in comparable results (Figures S3–5, Table S5), we used models based on logistic regression throughout this study.

**Figure 2:**
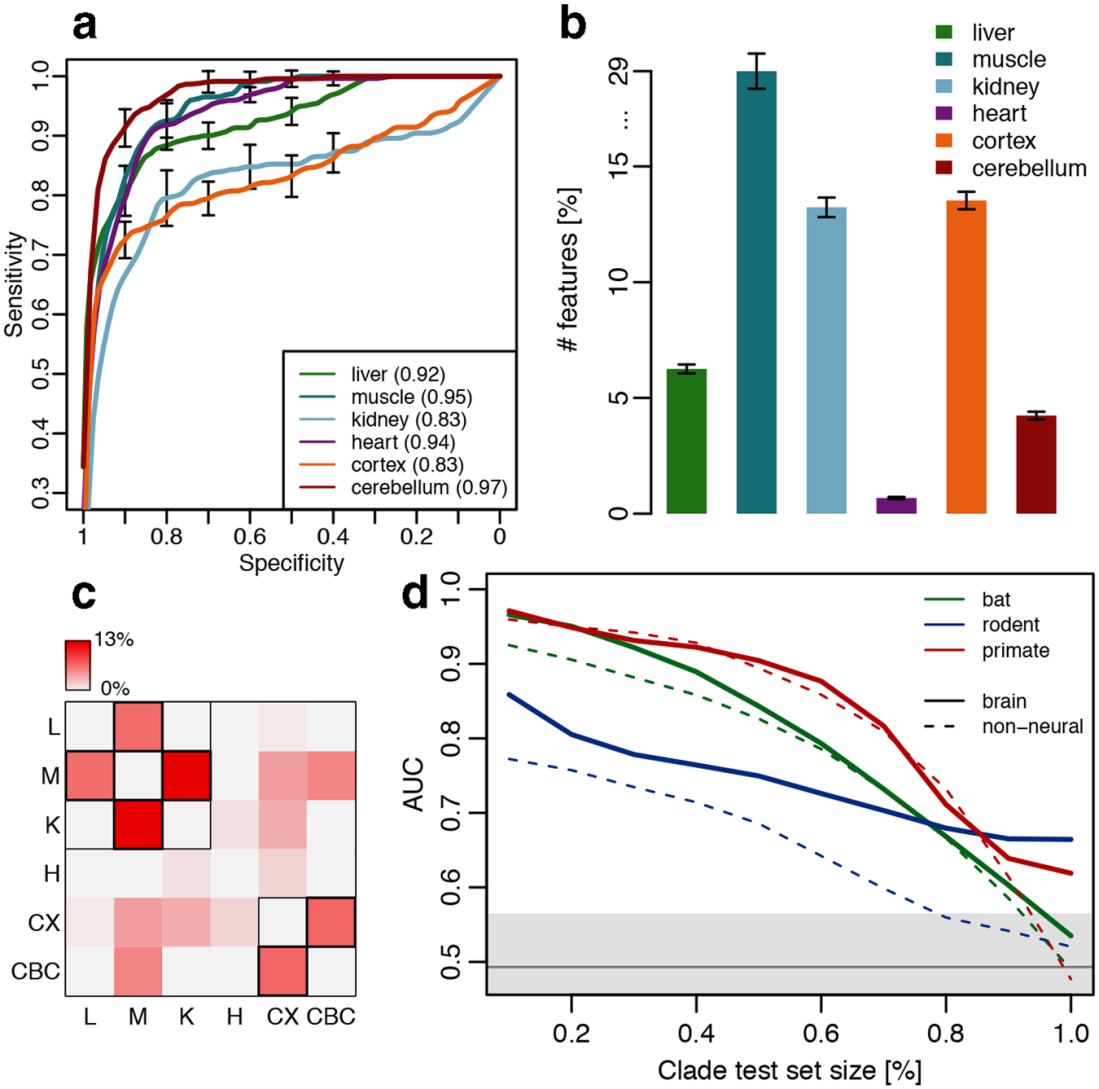
Long lifespan prediction. (**a**) Accuracy of predictive models in each tissue. (**b**) Proportion of lipid compounds identified as MLS predictors in each tissue. (**c**) Proportion of MLS predictors shared between tissues. Black borders indicate significant overlap (p < 0.05). (**d**) AUC of predictive models, constructed on training sets excluding an increasing proportion of randomly sampled individuals of one clade, with the models’ accuracy, tested on the clade individuals excluded from the training set. Identity of the test clade is indicated by the curve color. The horizontal gray line and shaded region represent mean and standard deviation of the random predictor accuracy calculated by randomizing the output variable 100 times.

Remarkably, in each of the six tissues, lipid concentrations identified the species with long MLS with an average accuracy of 0.91, and a maximum accuracy of 0.97, estimated as the area under the receiver operating characteristic curve (AUC) (Fig. 2a). Notably, the lipids identified as long MLS predictors were not predictive of species’ BMR, body mass and temperature, or other confounding factors when these were used as an output variable (Figure S6). The long MLS predictions were also robust with respect to uncertainties in the reported MLS of some of the species. We additionally assessed the robustness of the MLS predictors against age-related changes in lipid concentrations using 104 additional human samples from one brain tissue (cortex) and one non-neural tissue (heart) spanning the entire human postnatal lifespan (Table S6). Of the 14,104 and 26,105 compounds detected in cortex and heart, 75% and 25%, respectively, showed significant concentration changes with age (Table S7). Excluding these age-dependent lipids from our data did not decrease MLS prediction accuracy (Figure S7). Similarly, lipid concentration changes induced by postmortem delay^28^ showed no association with long MLS predictions (Table S8).

The proportion of lipids selected by the models as the long MLS predictors varied substantially among tissues, comprising 1% of the lipid compounds in heart, 4% in cerebellum, 7% in liver, 14% in kidney, 14% in brain cortex and 30% in skeletal muscle (Fig. 2b, Table S9). Thus, long lifespan markers are distributed over a larger portion of the lipidome in skeletal muscle, brain cortex, and kidney compared to heart, cerebellum and liver. Despite variation in the number of MLS predictive features among tissues, they overlapped significantly between brain cortex and cerebellum, as well as among three non-neural tissues: liver, muscle and kidney (Fig. 2c, Table S10). Thus, our MLS prediction models might rely on common lipid composition and pathways in these two tissue types.

The high performance of MLS prediction models based on lipid concentrations in the three mammalian clades suggests the presence of common markers of long lifespan shared across the clades. To inspect this further, we tested the models’ ability to predict MLS in a species from one clade based on the lipid information from the remaining two clades. Removal of an increasing number of individuals representing one clade from the training set resulted in a gradual reduction in prediction performance for this clade both in brain and in non-neural tissues (Figures S8 and 9). Still, the models based on brain data showed an above random performance even when all individuals of a given clade were removed from the training set (Fig. 2d). In other words, a model based solely on brain lipidome information from two clades, e.g. rodent and bats, is sufficient to make valid predictions about MLS for a species in the primate clade.

### Lipid predictors of long MLS

**Figure 3:**
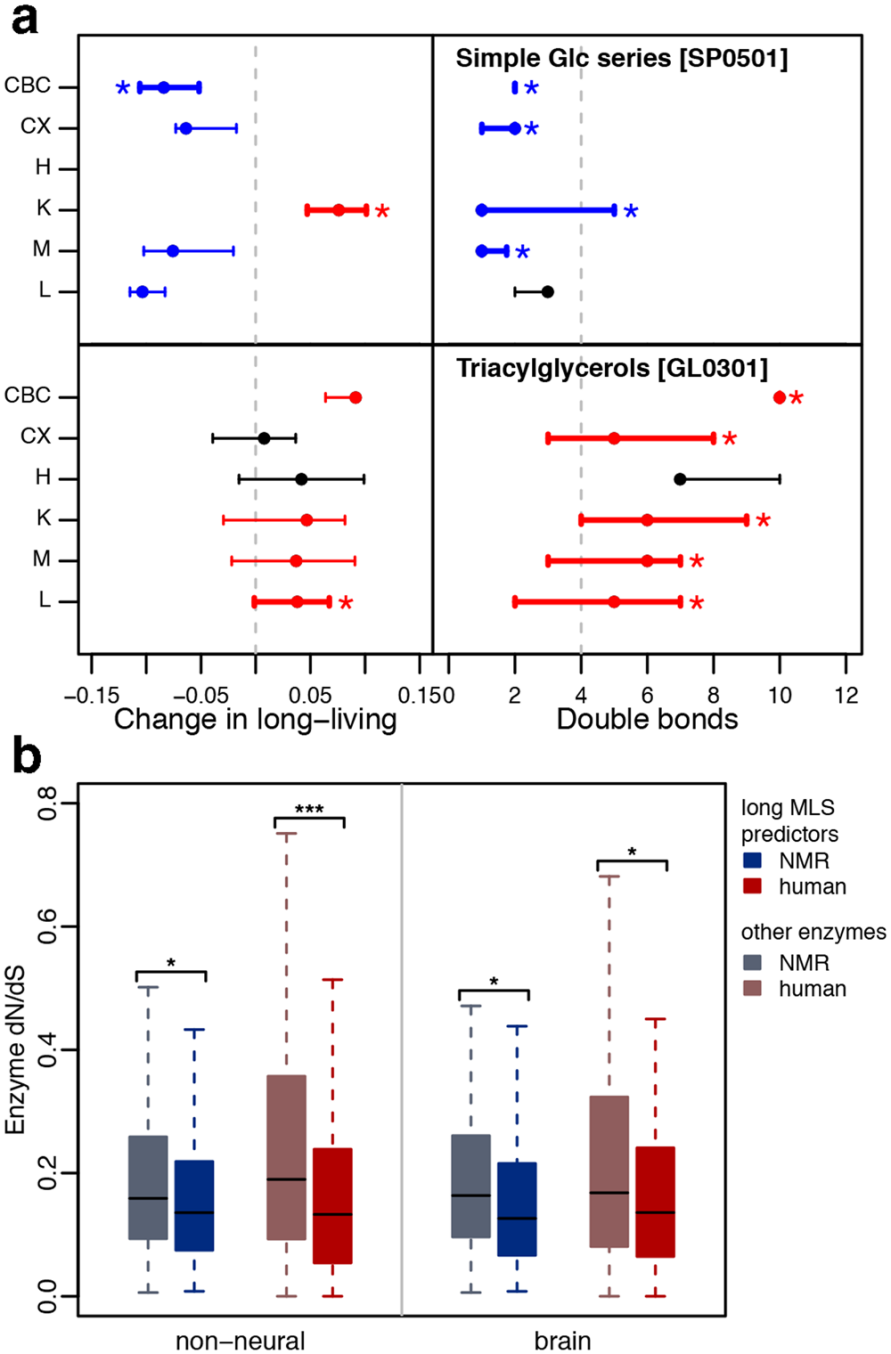
Long-lifespan related lipids. (**a**) Concentration level change and double bond distribution of long MLS predictors belonging to two lipid sub-classes in six tissues. Change in the concentration level is calculated as the difference between mean of the concentration levels in the long-living species and all other species in each of the clades. Left panel shows median concentration change of long MLS predictors belonging to simple Glc series and triacylglycerol lipid sub-classes in long-living species. The error bars show the 0.25 to 0.75 inter-quintile intervals. Significant concentration shift towards lower and higher values in long-living species is indicated by blue and red colors, respectively. Significant enrichment of MLS predictors among detected compounds in a given lipid sub-class is shown by thick lines and asterisks. The vertical dashed line shows the median concentration change for all long MLS predictors. Right panel shows the median double bond number and its 0.25 to 0.75 inter-quintile intervals for the same lipid sub-classes as in the left panel. Significantly higher or lower number of double bonds in a given lipid group is shown by red and blue color respectively, as well as thick lines and asterisks. The vertical dashed line shows the median number of double bonds in all long MLS predictors. (**b**) Enzyme DNA sequence variation. Distributions of the dN/dS values of the enzymes linked to the lipid predictors of long MLS (darker colors), and the enzymes linked to other lipids detected in our dataset (lighter colors), in brain and non-neural tissues (liver, muscle, and kidney) in the long-living species. Asterisks indicate p-value range *p < 0.05, **p < 0.01, ***p < 0.001.

Existence of common predictors of long lifespan suggests coordinated lipid concentration changes in long-living primates, rodents and bats. Indeed, changes in concentration of long lifespan predictors were positively and significantly correlated between clades, unlike changes in concentration of other lipids (permutation, p < 0.01) (Figure S10). Lipid predictors showing coordinated concentration change in long-living species of all three clades were enriched in 12 specific lipid classes, 24 sub-classes and 12 metabolic pathways in all tissues except heart (hypergeometric test, permutation, p < 0.05) (Table S11). What’s more, concentrations of long MLS predictors were significantly shifted towards either higher or lower values in the long-living species in at least one tissue in 11 of 12 enriched lipid classes, 17 of 24 sub-classes and 10 of 12 metabolic pathways (permutation, p < 0.05) (Table S11). Among them, triacylglycerols enriched in liver showed an increase in concentration levels in long-living species in almost all tissues; glycerophospholipids enriched in brain cortex were showing a decrease in concentration levels in most of the tissues; and sphingolipid metabolism pathway and several enriched classes of sphingolipids showed predominantly a decrease in concentration levels in all tissues except kidney (Fig. 3a, Figure S11). We inspected the relationship of the number of double bonds in lipid chains of the MLS predictors with their concentration levels in the long-living species and found several significant associations e.g. in sphingolipids, triacylglycerols, glycerolipids (Figure S11). However, no consistent pattern across all lipid classes and tissues could be observed. In order to inspect if the removal of lipids related to other confounding factors such as basal metabolic rate, body size, or body temperature could has affected these results, we performed the same analysis on the initial dataset before removal of the confounder-related lipids. A similarly inconsistent link between lipid concentration levels and double bond number was found among the enriched (Figures S12 and S13) as well as all lipid classes (Table S12) nevertheless the significant associations in sphingolipids, triacylglycerols, glycerolipids were confirmed. Overall, saturated lipids show higher correlation with species lifespan on average in all tissues except heart (Figure S14).

Several fatty acids were reported to show significant associations to longevity^18–25^. To verify their role in our data we manually searched and annotated fatty acids in our mass spectrometry measurements. We tested their concentration level difference between the long-living and other species and compared this difference to the distribution of the same difference across all other lipids in our dataset. The majority of fatty acids showed elevated concentration levels in the long-living species with several of them being significant in particular in the non-neural tissues. Notably, none of the saturated fatty acids showed significantly higher concentration levels in the long-living species (Table S13).

### Evolutionary processes leading to long MLS

The presence of common lipidome features associated with long lifespan in the three clades suggests the existence of a shared molecular mechanism associated with MLS in rodents, primates, and bats. To corroborate this finding, we searched for a possible evolutionary signature of such a mechanism. We examined amino acid substitution levels, measured as dN/dS ratios, for enzymes linked to the long MLS predictors using available genomic data: genomes of long-living rodent and primate – naked mole-rat and human, as well as their short-living counterparts – mouse and macaque. We found that, in long-living species, these enzymes showed consistently and significantly lower amino acid substitution levels compared to enzymes linked to other lipid compounds detected in our data (Figs 3b, S15,16, Tables S14 and S15). By contrast, short-living species did not show a significant reduction of amino acid substitution levels in all primate tissues and all but two rodent tissues (Figure S17). This shows that metabolic processes identified as related to lifespan by our MLS prediction models indeed experienced increased evolutionary pressure in long-living species compared to short-living species, in both rodent and primate clades.

**Figure 4:**
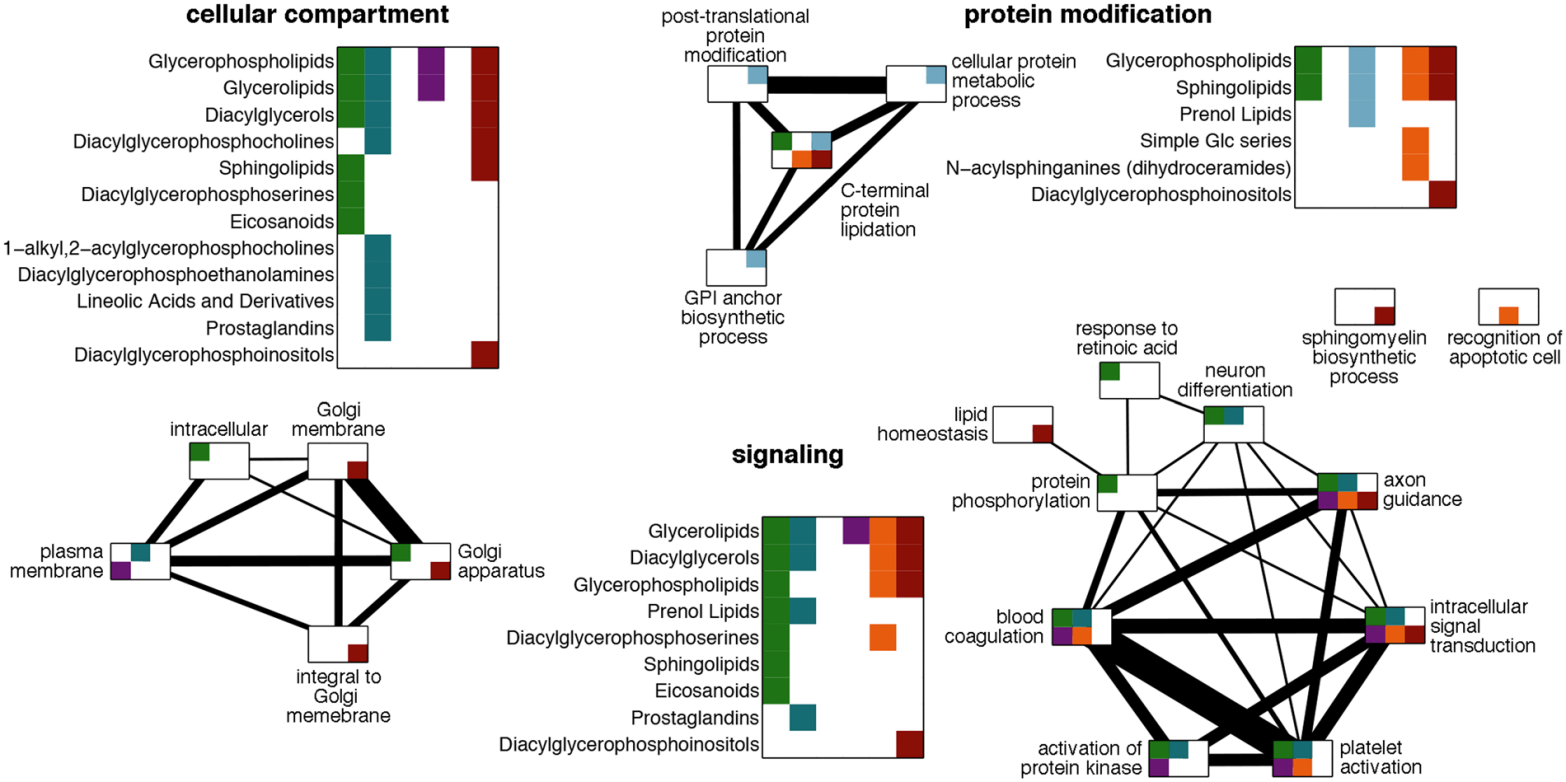
Functional modules. The network nodes represent GO terms significantly enriched in enzymes linked to the lipid predictors of long MLS, and having lower dN/dS values in the long-living species compared to the short-living species. The edges represent enzymes shared between terms with line width proportional to the number of shared enzymes: from 1 to 12. Colored squares within each GO term node indicate the tissue in which these enzymes are linked to the lipid predictors of long MLS: green – liver, dark blue – muscle, light blue – kidney, magenta – heart, orange – brain cortex, brown – cerebellum. Sub-classes of the lipid predictors of long MLS linked to the enzymes in each functional group are listed next to the corresponding network module. The colors indicate the tissues where they are used as predictors of long MLS, according to the same color code as the GO terms.

We next inspected the biological processes that involve enzymes linked to long MLS predictors, which additionally show a signature of increased conservation pressure in long-living species compared to their short-living clade counterparts. Analysis of Gene Ontology (GO) functional terms^29^ revealed a significant enrichment of the enzymes with evidence of higher negative selection in all six tissues, with several GO terms overrepresented in more than one tissue (Table S16). The resulting network based on enzymes shared between GO terms was composed of three distinct modules related to: (i) signaling processes, (ii) protein modification processes and (iii) cellular components (Fig. 4). The signaling module contained five GO terms universally present across tissues, including “intracellular signal transduction”, “activation of protein kinase”, “platelet activation”, and “axon guidance”, and four tissue-specific terms, including “neuron differentiation” and “protein phosphorylation”. The protein modification module contained four GO terms including “post-translational protein modification” and “C-terminal protein lipidation”. The cellular component module contained five GO terms including “Golgi apparatus components” and “plasma membrane” (Fig. 4). Long MLS predictors associated with these modules, in turn, belonged to specific lipid sub-classes. They included: glycerolipids, diacylglycerols, and glycerophospholipids, which were associated with the signaling and cellular compartment modules in multiple tissues; sphingolipids, associated with the protein modification module; diacylglycerophosphoinsitols, simple Glc series, and N-acylsphinganines, associated with biological processes specific to brain; and prenol lipids and prostaglandinds associated with processes specific to non-neural tissues (Tables S14 and S16). These lipid sub-classes intersected significantly with the ones significantly enriched in the long MLS predictors (permutation, p < 0.01) and showed common concentration levels shifts in long-living species (Table S11). Among them, lipids in the cellular compartment term showed lower concentration levels in the long-living species, while signaling and protein modification terms contained both lipid classes with elevated and reduced concentration levels in the long-living species.

## Discussion

Our results show that long lifespan is associated with distinct lipidome features shared across three mammalian clades. Lipid predictors of long MLS differ across tissues, but overlap within brain and non-neural tissue types. The long MLS predictors identified in brain are especially conserved among clades, allowing long-living species identification within a clade solely based on lipid predictors from the other two clades.

The existence of lipid predictors shared among the three clades further manifested itself in form of specific lipid classes, sub-classes and pathways up- or down-regulated within long-living species of all three clades. While little can be said about functional significance of these changes one potentially important feature stands out: the genomes of long-living primate and rodent species show increased evolutionary selection acting upon the amino acid sequences of enzymes linked to lipid predictors of long MLS. These enzymes cluster in specific functional categories associated with signaling and protein modification processes, as well as the corresponding cellular compartments: Golgi apparatus and plasma membrane.

Another interesting observation is the role of double bonds in lipid chain in relationship to long lifespan. In contrast to energy lipids, structural lipids, such as sphingolipids, the major constituent of cell membrane show higher saturation degree in long-living species presumably as means of protection from continuous oxidative stress (Figs 3a, S11). Lower number of double bonds in these lipids potentially represents a mechanism for maintaining their stability over the extended lifespans of respective species. The relationship between the number of double bonds and species lifespan is more complex for energy-related lipids. The observed higher concentration of unsaturated lipids such as triacylglycerols in long-living species compared to other species could be related to the efficiency of energy release of these lipids. As saturated lipids do not require creation of a double bond within the beta-oxidation process it allows them for an accelerated energy production. Therefore, comparatively slower energy production of the unsaturated energy lipids could be related to the slower metabolism characteristic of long-living animals.

The relationship between the number of lipid chain double bond and its concentration level in long-living species could not be established across tissues and lipid classes, however several significant observations were found (Figure S11). As lipid saturation was associated with lifespan by a number of studies^18–25^ to fully elucidate this mechanism of action specific tissue and lipid class examples should be investigated in detail and further validated experimentally.

The complex collection procedure of the large set of tissue samples used in our study did not allow us to make a comprehensive study of postmortem delay effect on our results. For this reason, we relied on a previously published data in which lipid degradation with postmortem time was extensively assessed^28^. We found a small and insignificant overlap of the long-lifespan predictors in our study with the postmortem delay-related lipids reported previously (Table S8). Therefore, in spite of the limited power of this method of assessing the effect of postmortem delay in our data, we conclude that it should not have a dramatic effect on the major results of this study.

In our approach, we were not able to discriminate between the causal lipidome determinants of a species’ MLS and its byproducts. Nonetheless, the existence of a defined signature for long MLS, shared among three mammalian clades, which was reflected not only by lipid concentrations, but also enzyme conservation and functional clustering, strongly suggests the presence of a distinct molecular mechanism controlling MLS variation among mammalian species. Identification of this mechanism may give us a unique opportunity to understand the determinants of lifespan duration and the nature of the ageing process.

## Materials and Methods

### Samples

Human samples were obtained from the NICHD Brain and Tissue Bank for Developmental Disorders at the University of Maryland, the Netherlands Brain Bank, and the Chinese Brain Bank Center (CBBC, http://cbbc.scuec.edu.cn, Wuhan, China). According to the protocol of these institutions, specific permission for brain autopsy and use of the brain tissue for research purpose was given by the donors or their relatives. Use of human autopsy tissue is considered non-human subject research and is IRB exempt under NIH guidelines. All subjects were defined as healthy with respect to the sampled tissue, by forensic pathologists at the corresponding tissue bank. All subjects suffered sudden death with no prolonged agony state.

Primate samples were obtained from Simian Laboratory Europe (SILABE) in Strasburg, research unit CNRS-MNHM 717 in Brunoy, France, German Primate Center (DPZ) in Goettingen, Max Planck Institute for Anthropology in Leipzig, Germany, Suzhou Experimental Animal Center in China, the Anthropological Institute and Museum of the University of Zürich-Irchel, Switzerland, and the Biomedical Primate Research Centre, the Netherlands. All non-human primates used in this study suffered sudden deaths for reasons other than their participation in this study and without any relation to the tissue used.

Rodent samples were obtained from the University of Rochester Biology Department, MDC Berlin, Department of Zoology and Entomology, University of Pretoria, and the animal center at the Shanghai Institute for Biological Sciences. All mice were from the C57/BL6 strain with no genetic modifications. Bat samples were obtained from Kunming Institute of Zoology of the Chinese Academy of Sciences. The use and care of the animals in this research was reviewed and approved by Biological Research Ethics Committee, Shanghai Institutes for Biological Sciences, Chinese Academy of Sciences. All animals were lawfully acquired and their retention and use were in every case in compliance with national and local laws and regulations, and in accordance with the Institute for Laboratory Animal Research (ILAR) Guide for Care and Use of Laboratory Animals.

Whenever possible, cortex samples were dissected from the anterior part of the superior frontal gyrus, a cortical region corresponding to Brodmann area 10 in humans. For smaller animals, a frontal part of the frontal cortex was taken. The CBC samples were dissected from the lateral part of the cerebellar hemispheres. For all brain samples we took special care to dissect gray matter only. The kidney samples were dissected from the renal cortex. Muscle tissue was dissected from thigh skeletal muscle, mainly from *M*. *vastus lateralis*. The heart samples were dissected from heart muscle tissue avoiding fat and heart valves. Liver tissue was dissected from the peripheral part of the liver lobes.

Each tissue sample was of approximately 20 mg of weight and was dissected from the frozen postmortem tissue on dry ice without thawing. The tissue samples were first powdered using a mortar and pestle cooled by liquid nitrogen and then prepared for the lipidome extraction procedure.

### MS sample preparation and measurements

Lipids were extracted from frozen tissue powder by solution of methanol, methyl-*tert*-butyl-ether 1:3 (v/v) according to Giavalisco *et al.*^30^. Briefly, 20 mg of frozen powdered tissue material was resuspended in 1 ml extraction solution containing 0.5 μg of corticosterone, 1.5 μg of 1,2-diheptadecanoyl-*sn*-glycero-3-phosphocholine (Avanti Polar Lipids, 850360P, City State), 0.5 μg of 13C sorbitol and 0.25 μg of ampicillin. The samples were incubated for 10 min at 4 °C on an orbital shaker. This step was followed by ultrasonication in an ice-cooled bath-type sonicator for 10 min. To separate the organic from the aqueous phase 500 μl of a H_2_O:methanol mix (3:1(v/v) was added to the homogenate, vortexed and centrifuged (5 min; 14,000 g). Finally, 500 μl of the supernatant (MTBE phase containing lipid compounds) was collected to a fresh 1.5 ml Eppendorf tube for lipid analysis. This aliquot was concentrated to complete dryness in a speed vacuum at room temperature.

Prior to analysis the dried pellets were re-suspended in 500 µL acetonitrile:isopropanol (7:3) (v:v)), ultrasonicated, and centrifuged (5 min; 14,000 g). 200 µL of the cleared supernatant was transferred to fresh glass vials, of which 2 µL were loaded onto a UPLC system (Acquity, Waters) equipped with a C_8_ reverse phase column (100 mm × 2.1 mm × 1.7 µm particles, Waters). In addition to the individual tissue samples we measured mixtures of tissue samples (pooled samples). These pools were prepared by mixing 10 µL of each individual sample after resuspension of the dried lipid pellet. Pooled samples were measured after every 40^th^ sample, providing us information on system performance (sensitivity and retention time consistency), sample reproducibility and compound stability over the time of the MS-based analysis.

The mobile phase contained water (UPLC MS grade, BioSolve) with 10 mM NH_4_ac, 0.1% acetic acid (Buffer A), and acetonitrile/isopropanol (7:3, UPLC grade Biosolve) containing 10 mM NH_4_ac, 0.1% acetic acid (Buffer B). The flow rate of the system was set to 400 µL/min, and the gradient to: 1 min 45% A, 3 min linear gradient from 45% A to 35% A, 8 min linear gradient from 25% A to 11% A, 3 min linear gradient from 11% A to 1% A. After washing the column for 3 min with 1% A, the buffer is set back to 45% A, and the column is re-equilibrated for 4 min (22 min total run time).

The mass spectra were acquired using an Exactive mass spectrometer (Thermo-Fisher, Bremen, Germany). The spectra of the pooled samples were recorded using altering full scan and all ion fragmentation scan mode, covering a mass range from 100–1500 m/z. The individual samples were measured using two time segments where the measurements were performed either in negative (0–13 min) or in positive (13–20 min) ionization mode. This strategy allowed us to reduce the number of measurements by 50%. The resolution for all measurements was set to 25,000 providing 5 scans per second, restricting the loading time of the orbitrap cell to a maximum of 100 ms. The capillary voltage was 3 kV with a sheath gas flow value of 60, an auxiliary gas flow of 35, and a capillary temperature of 150 °C. The drying gas in the heated electro spray source was 350 °C, while the skimmer voltage and the tube lens were 25 V and 130 V, respectively. The spectra were recorded from 0 min to 20 min of the UPLC gradients.

### Peak extraction and alignment

Peaks were extracted using the Progenesis QI metabolomics software (Version 1.0.5165, Nonlinear Dynamics, New Castle, UK) allowing only the detection of a single isotope (M + H)^+^ in positive- and (M − H)^−^ in negative ionization mode. The software automatically detects the different isotope peaks and clusters them together while reporting the summed intensity under the monoisotopic mass retention time feature. Accordingly we define as a peak, throughout this manuscript, as an individual mass trace of the first isotope. In a later step the different, possible adducts of each compound class were matched, as described in below to remove the redundancy from the peak annotation.

### Normalization

All peak concentration values were normalized by the internal standard (IS) PC 34:0 concentration levels and log transformed. Samples with IS concentration levels outside of a 2.5 standard deviation range around the mean of that IS in the tissue were removed from the dataset.

### Peak annotation

We used mass searches with a mass tolerance of 10 ppm, allowing adducts listed in Table S18 in the positive and negative ionization modes^31^. We tested a lower cutoff for database search of 5 ppm and obtained qualitatively the same results. The obtained m/z values were next searched against the LIPID MAPS^27^ database for the initial annotation of the positive and negative ionization mode lipid datasets. For each lipid class we defined a list of possible adducts, Table S18, used in this step of data base search. In the second step, all the remaining, less common, adducts were used in an additional database search to test the support of the database matches found in the first step of the annotation procedure (Table S19). Next, peaks showing a correlation >0.7, a difference in RT of <0.05 and matched to the same lipid compound in the database search were merged together. The average concentration level of the merged peaks in all samples was used in further analysis. This procedure resulted in merging 427–880 peaks in different tissues into composite peaks of size up to 6. As a result, the datasets contained between 5,313 and 13,067 compounds in different tissues and ionization modes and between 13,089 (cerebellum) and 20,669 (heart) compounds in both ionization modes together.

We used LIPID MAPS and HMDB database annotation to assign each identified compound to its lipid class and subclass. We tested the overrepresentation of lipid classes and subclasses in a given compound group using a hypergeometric test corrected for multiple testing by random sampling of the same number of lipid compounds from our dataset 1000 times.

To determine the number of double bonds of the compounds assigned to each peak we used their InChI identifiers^32^.

### Confounder correlation search

We searched the literature^33,34^ for the reported species basal metabolic rate (BMR), body temperature, diet, body mass, and hibernation information (Table S20). In addition, we collected individual sex and age information (Table S1). The relation of lipid concentration levels to these factors was tested based on (a) t-test for sex and hibernation factors, (b) analysis of variance (ANOVA) for diet factors, (c) linear regression analysis for BMR, body temperature, body mass, and age factors. The resulting p-values were further corrected for multiple testing using permutations. Lipid compounds showing significant (p < 0.01) relationship with any of the factors were removed from the analysis (Table S3).

Previous work^28^ has shown that concentrations of less than 8% of lipids detected in various primate tissues are affected by postmortem delay (PMD). We found no significant association between lipids showing these PMD-associated changes and the long MLS predictors (Table S8). Furthermore, samples collected from long-living species in our dataset are characterized by different PMD: no PMD for naked mole-rats, <5 h for bats, and up to 50 h PMD for humans. Thus, long MLS predictors shared among the three clades cannot be due to the effect of PMD.

In the tests requiring matching lipid peaks between datasets, including the time-series and PMD measurements, cutoffs of 10 ppm of the mass to charge ratio and 0.1 sec RT were used. Age-related lipid change was assessed using a polynomial function of up to 3^rd^ degree fit to each lipid compound measurement (F-test, p < 0.01).

### Classifier construction

We used logistic regression with elastic net penalty to construct binary classifiers distinguishing the long-living species from other species in the dataset. The α parameter of the classifier was chosen based on the classification accuracy in 10 × 10 cross-validation runs. Classifier accuracy was assessed through cross-validation, for the functional analysis the model was retrained on all sample data using the α parameter value selected in the cross-validation procedure.

### Other models

For comparison, we implemented a feature selection procedure for a support vector machine (SVM) classifier according to^35^. In every cross-validation run, iteratively, 1% of the initial number of features with the lowest coefficient in the linear SVM was removed and the model was retrained on the remaining features until there were no more than 10 features left. Out of all numbers of features tested in the iterative procedure, the best performing one was chosen and the model accuracy was assessed as the average across the cross-validation runs for the chosen number of features. For model comparison, the SVM was retrained using all sample data in the iterative procedure until the chosen number of features was reached.

In the same way as the logistic regression model we constructed a linear regression model with elastic net penalty. For the response variable in this model we used lifespan of a species normalized to the maximum lifespan of a species within each clade.

### Individual clade prediction test

In order to inspect the robustness of the long lifespan classifier based on incomplete clade information, we performed a test of eliminating an increasing proportion of a clade individuals from the training set and using them in the test set. In this procedure we randomly sampled an increasing proportion of clade individuals from 10% to 90% in 10%-steps, 100 times in every step. In each randomization step, after calculating prediction models for all individual tissues, tissue datasets were merged into: a non-neural dataset including liver, muscle and kidney, and a brain dataset including cortex and cerebellum. The previously sampled training and test sets in the individual tissues were merged to test the prediction accuracy of models based on the merged data. Prediction accuracy of the models based on limited clade data was estimated as the average of all 100 sampling trials done for each elimination step.

Lipid datasets in the randomization procedure were merged based on the coefficients of lipid peaks in the individual tissue models. Individual tissue lipid data were first sorted according to the coefficients in the individual-tissue models, and according to the difference in the concentration levels between long- and short-living individuals in the training set. Then, peaks with zero coefficients and lowest difference between the long- and short-living individuals were removed in order to create datasets of the same sizes. Finally, the datasets were merged according to the predefined order.

### Genome analysis

We used genomes of primate species (human, rhesus macaque and marmoset) and rodent species (naked mole-rat and mouse). Orthologous gene information for 908 lipid enzymes identified based on their link to the lipids in our dataset^27,36^ was downloaded from NCBI Gene database (ftp://ftp.ncbi.nih.gov/gene/DATA/gene_group.gz) and concatenated into Table S17. We used *muscle*^37^ to align exonic sequences (consensus CDS only, according to NCBI Nucleotide database) of corresponding RNAs annotated in NCBI Gene database ftp://ftp.ncbi.nih.gov/gene/DATA/gene2refseq.gz, ‘reviewed’ status only), in two sets of species: (1) human, rhesus macaque and marmoset as an outgroup; (2) naked mole-rat, mouse and human as an outgroup. We used *codeml* (with all zero parameters except CodonFreq = 2, model = 2, kappa = 2, omega = 2, fix_alpha = 1, ncatG = 4)^38^ to calculate dN/dS ratios for all tree branches separately (Table S17).

We next compared the distribution of the dN/dS ratios of enzymes linked to the long MLS predictors and enzymes linked to other lipids in our dataset. For each enzyme, we calculated the proportion of the number of linked long MLS predictors to the number of all lipids this enzyme is linked to. We defined as lifespan-related the enzymes with top 30% (top 25% in heart and top 35% in non-neural tissues) proportions of the long MLS predictors in a given tissue. The dN/dS ratios of these lifespan-related enzymes were compared to the dN/dS of other lipid enzymes.

## Electronic supplementary material

Supplementary Information

Supplementary Information

Supplementary Information

Supplementary Information

Supplementary Information

Supplementary Information

Supplementary Information

Supplementary Information

Supplementary Information

Supplementary Information

Supplementary Information

Supplementary Information

Supplementary Information
