# Publisher Correction: Lipidome determinants of maximal lifespan in mammals

**DOI:** 10.1038/s41598-019-43122-9

**Published:** 2019-05-01

**Authors:** Katarzyna Bozek, Ekaterina E. Khrameeva, Jane Reznick, Damir Omerbašić, Nigel C. Bennett, Gary R. Lewin, Jorge Azpurua, Vera Gorbunova, Andrei Seluanov, Pierrick Regnard, Fanelie Wanert, Julia Marchal, Fabien Pifferi, Fabienne Aujard, Zhen Liu, Peng Shi, Svante Pääbo, Florian Schroeder, Lothar Willmitzer, Patrick Giavalisco, Philipp Khaitovich

**Affiliations:** 1 CAS Key Laboratory of Computational Biology, CAS-MPG Partner Institute for Computational Biology, 320 Yue Yang Road, Shanghai, 200031 China; 2 Max Planck Institute for Evolutionary Anthropology, Deutscher Platz 6, 04103 Leipzig, Germany; 3 Skoltech Center for Computational and Systems Biology, Skolkovo Institute for Science and Technology, Novaya st. 100, Skolkovo, 143025 Russia; 4 Institute for Information Transmission Problems, Russian Academy of Sciences, Bolshoy Karetny per. 19/1, Moscow, 127051 Russia; 5 Max-Delbrück Center for Molecular Medicine, Department of Zoology and Entomology, University of Pretoria, Pretoria, Republic of South Africa South Africa; 6 Max-Delbrück Center for Molecular Medicine, Robert-Rössle-Str. 10, 13092 Berlin, Germany; 7 Department of Zoology and Entomology, University of Pretoria, Pretoria, Republic of South Africa South Africa; 8 University of Rochester, 434 Hutchison Hall, Rochester, NY 14627-0211 USA; 9 SILABE, Primatology Center, University Louis Pasteur, Strasbourg, 4 Rue Blaise Pascal, 67070 France; 10 UMR 7179/CNRS-MNHN, 1 avenue du Petit Chateu, 91800 Brunoy, France; 11 State Key Laboratory of Genetic Resources and Evolution, Kunming Institute of Zoology, Chinese Academy of Sciences, 32 Jiaochang Dong Lu, Kunming, Yunnan 650223 China; 12 Max Planck Institute for Molecular Plant Physiology, Am Mühlenberg 1, 14476 Potsdam, Germany; 13 Present Address: Okinawa Institute of Science and Technology Graduate University, 1919-1 Tancha, Onna-son, Kunigami-gun, Okinawa, 904-0495 Japan

Correction to: *Scientific Reports* 10.1038/s41598-017-00037-7, published online 31 January 2017

Twelve Supplementary Tables were omitted from the original version of this Article. This has been corrected in the HTML version of the Article; the PDF version was correct at time of publication.

