# Supplementary material for: Lipidome determinants of maximal lifespan in mammals
Source: Sci Rep. 2017 Jan 31;7:5. doi: 10.1038/s41598-017-00037-7 (PMC5428381; doi:10.1038/s41598-017-00037-7)
Supplement: Supplementary file 1 — Supplementary Information [file 41598_2017_37_MOESM1_ESM.pdf]

Lipidome determinants of maximal lifespan in mammals.

Katarzyna Bozek, Ekaterina E. Khrameeva, Jane Reznick, Damir Omerbašić, Nigel C. Bennett, Gary R. Lewin, Jorge Azpurua, Vera Gorbunova, Andrei Seluanov, Pierrick Regnard, Fanelie Wanert, Julia Marchal, Fabien Pifferi, Fabienne Aujard, Zhen Liu, Peng Shi, Svante Pääbo, Florian Schroeder, Lothar Willmitzer, Patrick Giavalisco, Philipp Khaitovich

Supplemental Material

List of Supplemental Figures

Figure S1. MLS relationship to other factors.

Figure S2. Proportion of detected lipid compounds showing concentration variation significantly associated with one of the confounding factors.

Figure S3. Accuracy of predictive models based on a support vector machine (SVM).

Figure S4. Accuracy of linear models of MLS.

Figure S5. Accuracy of predictive models based on logistic regression.

Figure S6. Accuracy of MLS models in predicting species BMR, body mass and temperature.

Figure S7. Accuracy of prediction models trained on lipid compounds not showing age-related change.

Figure S8. Accuracy of predictive models constructed on training sets excluding an increasing proportion of randomly sampled individuals of one clade.

Figure S9. AUC of predictive models presented in Figure S8.

Figure S10. Correlation of the concentration level change in long-living species of different clades.

Figure S11. Concentration level change in long-living species and double bond distribution of long MLS predictors belonging to different lipid sub-classes.

Figure S12. Concentration level change in long-living species and double bond distribution of long MLS predictors in the full lipidome dataset before removal of the peaks potentially related to confounders.

Figure S13. Concentration level change in long-living species and double bond distribution of long MLS predictors in the partial lipidome dataset after removal of the peaks potentially related to all confounders except the body size.

Figure S14. Correlation of the lipid concentration level and species lifespan of the saturated and unsaturated lipids.

Figure S15. Distribution of the dN/dS values in the long-living species of the enzymes linked to the long MLS predictors, and the enzymes linked to other lipid compounds detected in our data.

Figure S16. As in Figure 3: Distributions of the dN/dS values of the lipid enzymes in the long-living species.

Figure S17. As in Figure 3: distribution of the dN/dS values of the lipid enzymes in the short-living species.

Supplemental Figures

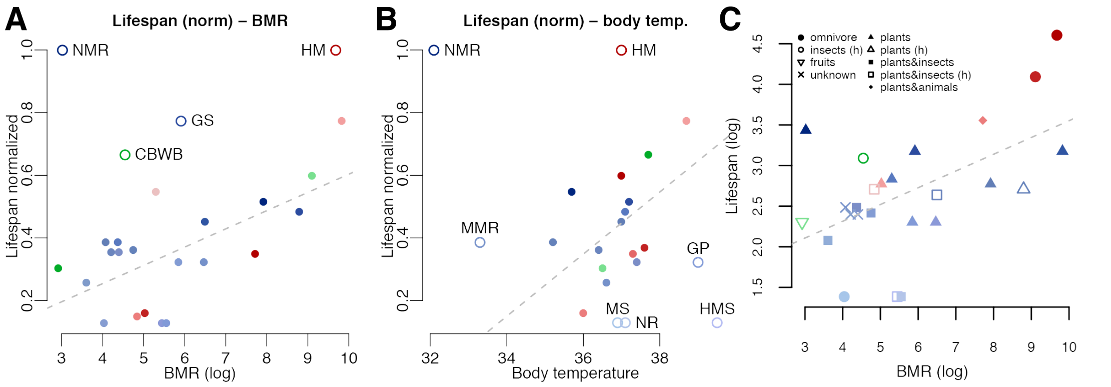

Figure S1. MLS relationship to other factors. (**A-B**) Relationship between normalized MLS and BMR or body temperature. Colors and symbols as in Figure 1D. (**C**) Relationship between actual MLS and BMR, diet and hibernation ability. Both MLS and BMR are plotted in log2-scale. Species diet and capacity to hibernate (h) is indicated by the symbols. Colors correspond to species colors in Figure 1D. NMR – naked mole rat, HM – human, GS – grey squirrel, CBWB – common bent-winged bat, MMR – mashona mole rat, RBFB – rickett's big-footed bat, CHB – chinese horseshoe bat, GP – guinea pig, MS – mouse, NR – norway rat, HMS – hamster.

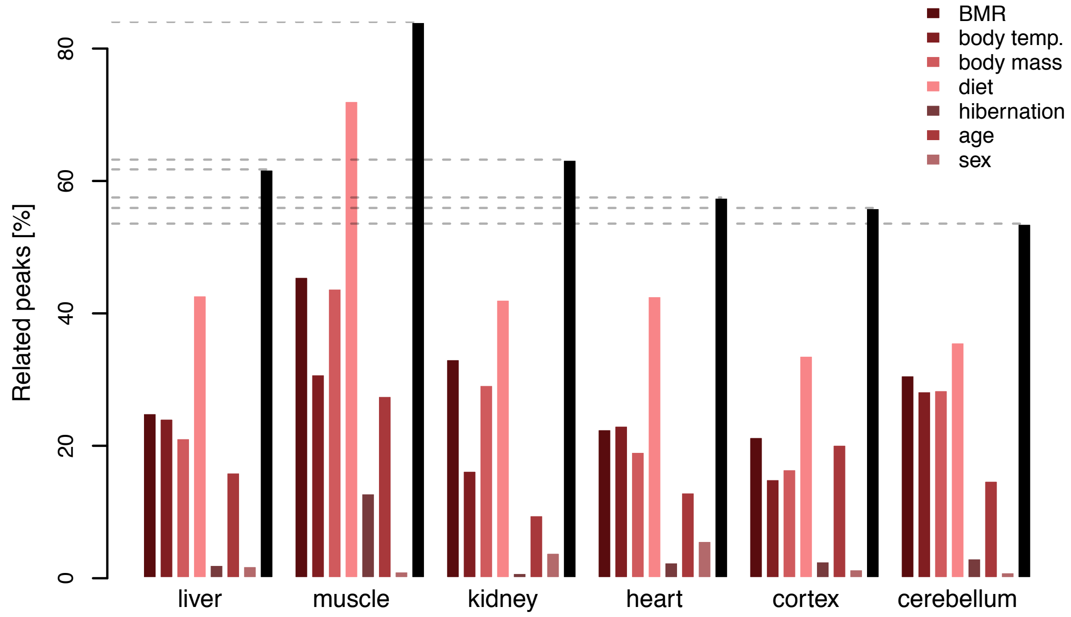

Figure S2. Proportion of detected lipid compounds showing concentration variation significantly associated with one of the confounding factors. Black bars indicate the proportion of compounds associated with any of the factors in each tissue.

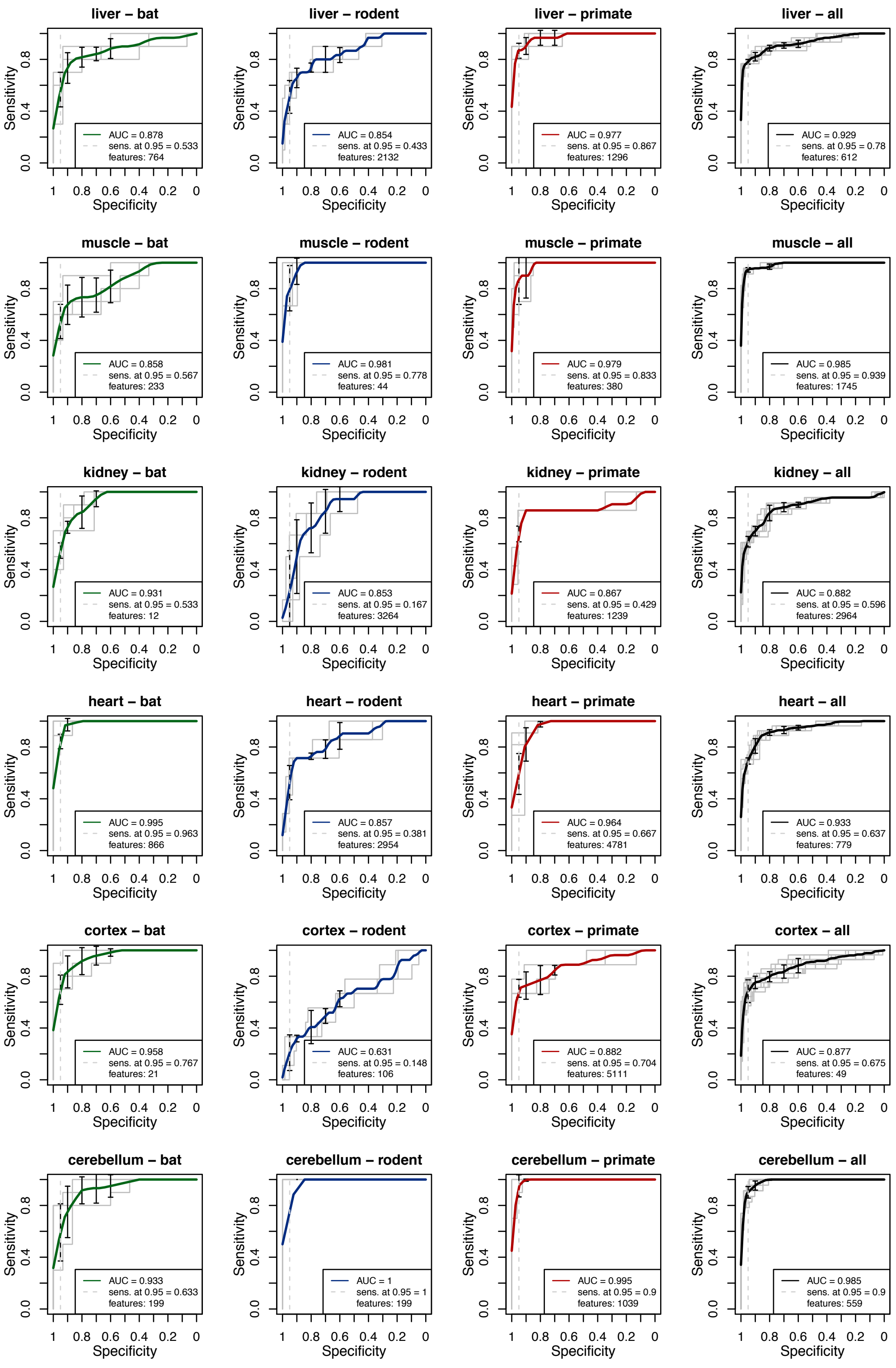

Figure S3. Accuracy of predictive models based on a support vector machine (SVM). The accuracy was tested in 10x10 cross validation. For more comprehensive comparison with other methods we tested the predictors in all clades separately and in all clades combined (last column). AUC and sensitivity at the 0.95 specificity thresholds were used as prediction accuracy measures. Average number of features selected in each cross validation run is indicated. Sensitivity is shown on the y-axis, specificity on the x-axis.

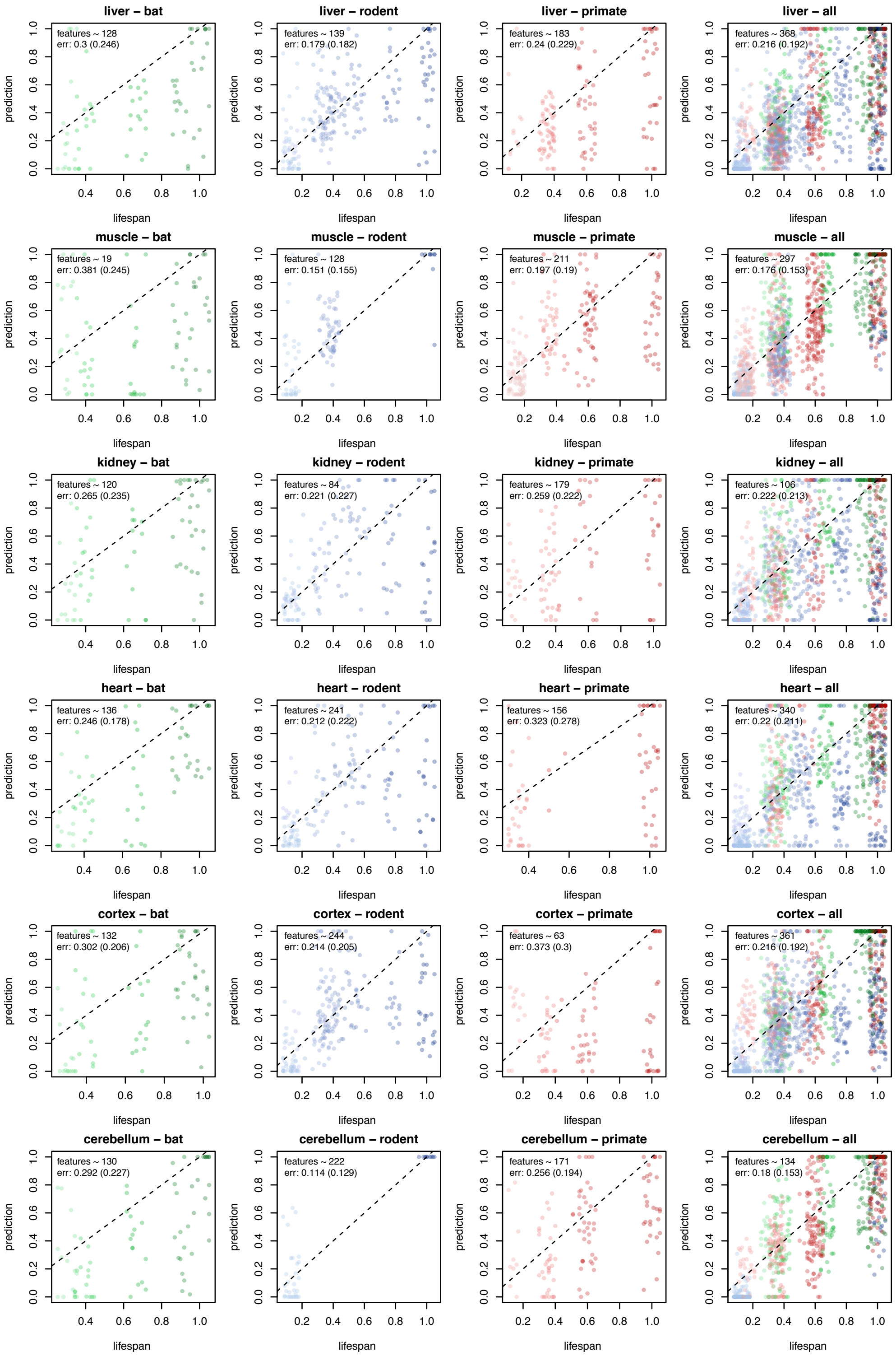

Figure S4. Accuracy of linear models of MLS. The accuracy was tested in 10x10 cross validation. Error was measured as the absolute difference between prediction and the MLS, mean and standard deviation of error in all cross validation runs is shown. Predictions of all cross validation runs are presented, colors correspond to clades: green – bats, blue – rodents, red – primates. Average number of features selected in each cross validation run is indicated.

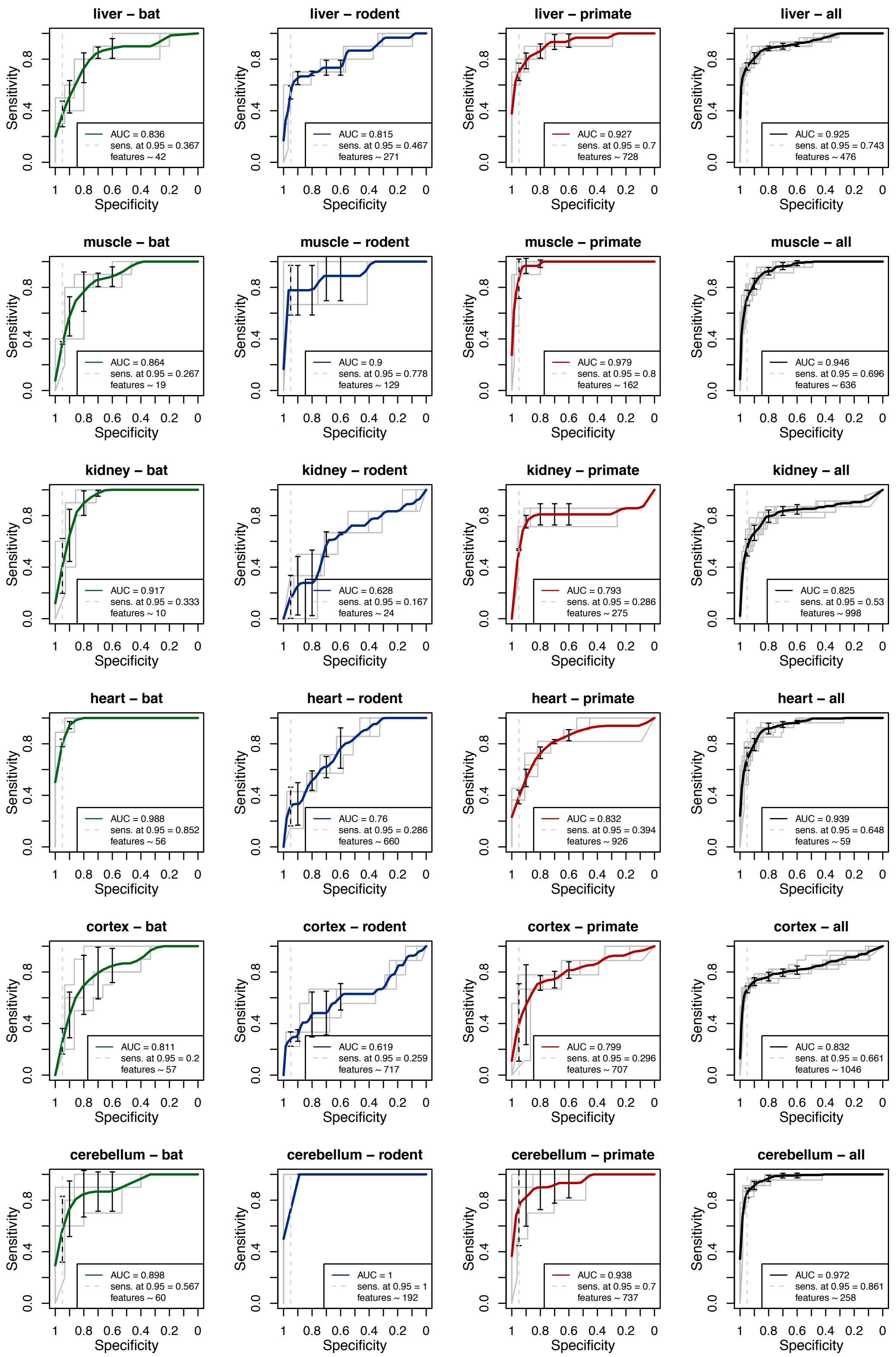

Figure S5. Accuracy of predictive models based on logistic regression. The accuracy was tested in 10x10 cross validation. Similar to above, the predictors in all clades separately, and all clades combined, were tested. Average number of features selected in each cross validation run is indicated. Sensitivity is shown on the y-axis, specificity on the x-axis.

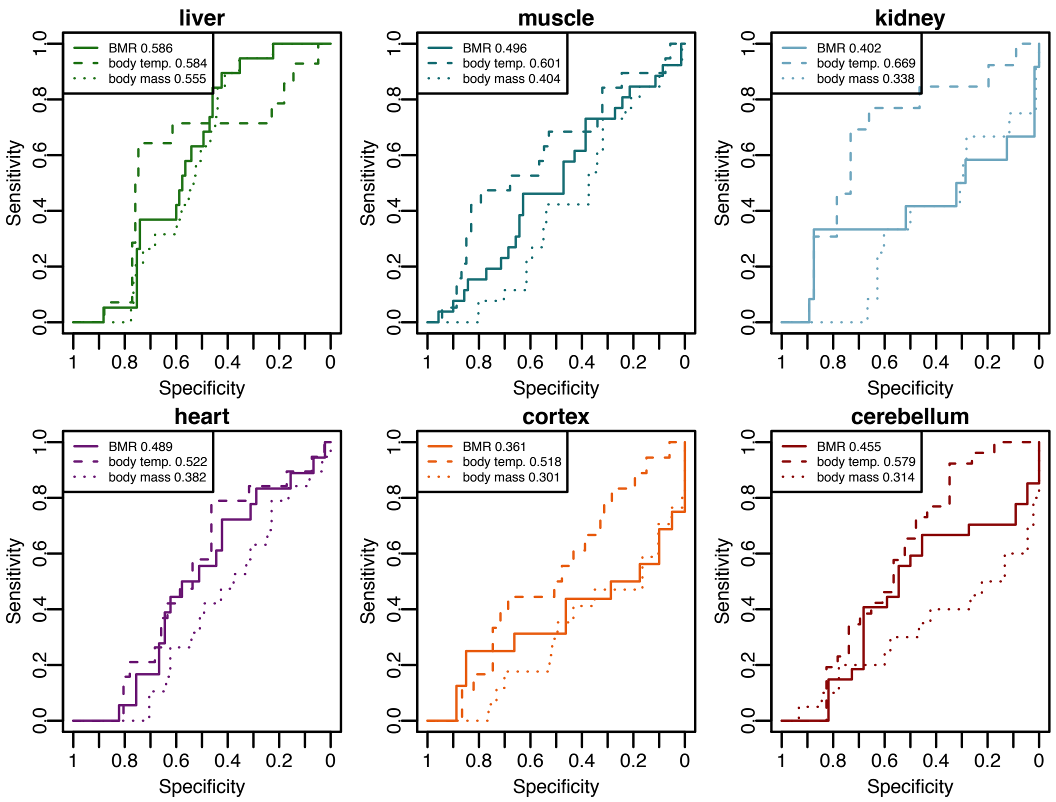

Figure S6. Accuracy of MLS models in predicting species BMR, body mass and temperature. Human samples were removed from this test as they represented the most extreme values of these parameters. Sensitivity is shown on the y-axis, specificity on the x-axis.

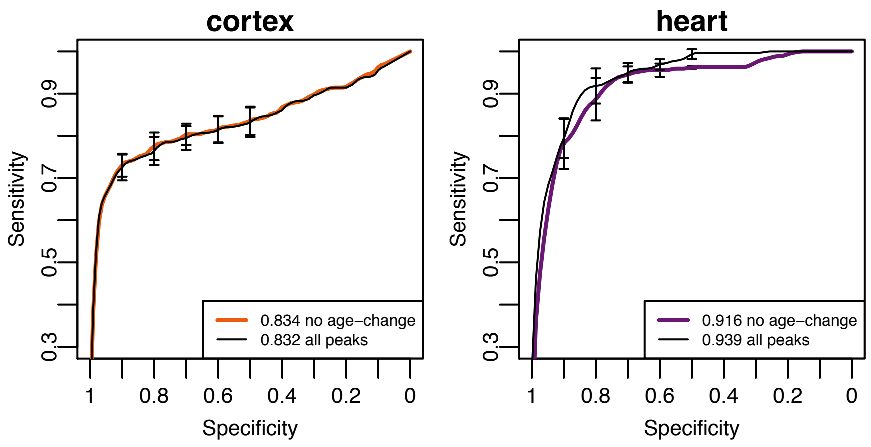

Figure S7. Accuracy of prediction models trained on lipid compounds not showing age-related change. For both heart and cortex, excluding the age-related lipids did not affect the prediction accuracy of the models.

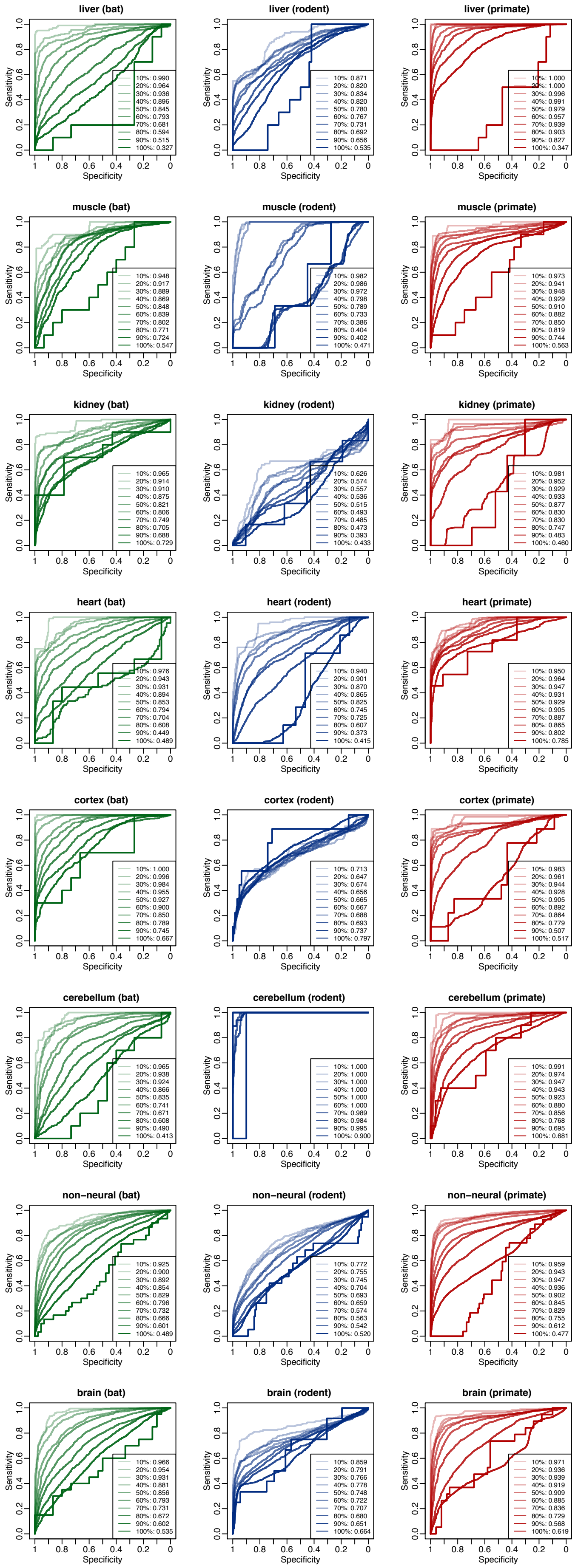
liver (bat)
10%: 0.990
20%: 0.964
30%: 0.936
40%: 0.896
50%: 0.845
60%: 0.793
70%: 0.681
80%: 0.594
90%: 0.515
100%: 0.327
liver (rodent)
10%: 0.871
20%: 0.820
30%: 0.834
40%: 0.820
50%: 0.780
60%: 0.767
70%: 0.731
80%: 0.692
90%: 0.656
100%: 0.535
liver (primate)
10%: 1.000
20%: 1.000
30%: 0.996
40%: 0.991
50%: 0.979
60%: 0.957
70%: 0.939
80%: 0.903
90%: 0.827
100%: 0.347
muscle (bat)
10%: 0.948
20%: 0.917
30%: 0.889
40%: 0.869
50%: 0.848
60%: 0.839
70%: 0.802
80%: 0.771
90%: 0.724
100%: 0.547
muscle (rodent)
10%: 0.982
20%: 0.986
30%: 0.972
40%: 0.798
50%: 0.789
60%: 0.733
70%: 0.386
80%: 0.404
90%: 0.402
100%: 0.471
muscle (primate)
10%: 0.973
20%: 0.941
30%: 0.948
40%: 0.929
50%: 0.910
60%: 0.882
70%: 0.850
80%: 0.819
90%: 0.744
100%: 0.563
kidney (bat)
10%: 0.965
20%: 0.914
30%: 0.910
40%: 0.875
50%: 0.821
60%: 0.806
70%: 0.749
80%: 0.705
90%: 0.688
100%: 0.729
kidney (rodent)
10%: 0.626
20%: 0.574
30%: 0.557
40%: 0.536
50%: 0.515
60%: 0.493
70%: 0.485
80%: 0.473
90%: 0.393
100%: 0.433
kidney (primate)
10%: 0.981
20%: 0.952
30%: 0.929
40%: 0.933
50%: 0.877
60%: 0.830
70%: 0.830
80%: 0.747
90%: 0.483
100%: 0.460
heart (bat)
10%: 0.976
20%: 0.943
30%: 0.931
40%: 0.894
50%: 0.853
60%: 0.794
70%: 0.704
80%: 0.608
90%: 0.449
100%: 0.489
heart (rodent)
10%: 0.940
20%: 0.901
30%: 0.870
40%: 0.865
50%: 0.825
60%: 0.745
70%: 0.725
80%: 0.607
90%: 0.373
100%: 0.415
heart (primate)
10%: 0.950
20%: 0.964
30%: 0.947
40%: 0.931
50%: 0.929
60%: 0.905
70%: 0.887
80%: 0.865
90%: 0.802
100%: 0.785
cortex (bat)
10%: 1.000
20%: 0.996
30%: 0.984
40%: 0.955
50%: 0.927
60%: 0.900
70%: 0.850
80%: 0.789
90%: 0.745
100%: 0.667
cortex (rodent)
10%: 0.713
20%: 0.647
30%: 0.674
40%: 0.656
50%: 0.665
60%: 0.667
70%: 0.688
80%: 0.693
90%: 0.737
100%: 0.797
cortex (primate)
10%: 0.983
20%: 0.961
30%: 0.944
40%: 0.928
50%: 0.905
60%: 0.892
70%: 0.864
80%: 0.779
90%: 0.507
100%: 0.517
cerebellum (bat)
10%: 0.965
20%: 0.938
30%: 0.924
40%: 0.866
50%: 0.835
60%: 0.741
70%: 0.671
80%: 0.608
90%: 0.490
100%: 0.413
cerebellum (rodent)
10%: 1.000
20%: 1.000
30%: 1.000
40%: 1.000
50%: 1.000
60%: 1.000
70%: 0.989
80%: 0.984
90%: 0.995
100%: 0.900
cerebellum (primate)
10%: 0.991
20%: 0.974
30%: 0.947
40%: 0.943
50%: 0.923
60%: 0.880
70%: 0.856
80%: 0.768
90%: 0.695
100%: 0.681
non−neural (bat)
10%: 0.925
20%: 0.900
30%: 0.892
40%: 0.854
50%: 0.829
60%: 0.796
70%: 0.732
80%: 0.666
90%: 0.601
100%: 0.489
non−neural (rodent)
10%: 0.772
20%: 0.755
30%: 0.745
40%: 0.704
50%: 0.693
60%: 0.659
70%: 0.574
80%: 0.563
90%: 0.542
100%: 0.520
non−neural (primate)
10%: 0.959
20%: 0.943
30%: 0.947
40%: 0.936
50%: 0.902
60%: 0.845
70%: 0.829
80%: 0.755
90%: 0.612
100%: 0.477
brain (bat)
10%: 0.966
20%: 0.954
30%: 0.931
40%: 0.881
50%: 0.856
60%: 0.793
70%: 0.731
80%: 0.672
90%: 0.602
100%: 0.535
brain (rodent)
10%: 0.859
20%: 0.791
30%: 0.766
40%: 0.778
50%: 0.748
60%: 0.722
70%: 0.707
80%: 0.680
90%: 0.651
100%: 0.664
brain (primate)
10%: 0.971
20%: 0.936
30%: 0.939
40%: 0.919
50%: 0.909
60%: 0.885
70%: 0.836
80%: 0.729
90%: 0.568
100%: 0.619
Sensitivity
Specificity

Figure S8. Accuracy of predictive models constructed on training sets excluding an increasing proportion of randomly sampled individuals of one clade. Accuracy for each tissue and test clade is shown.

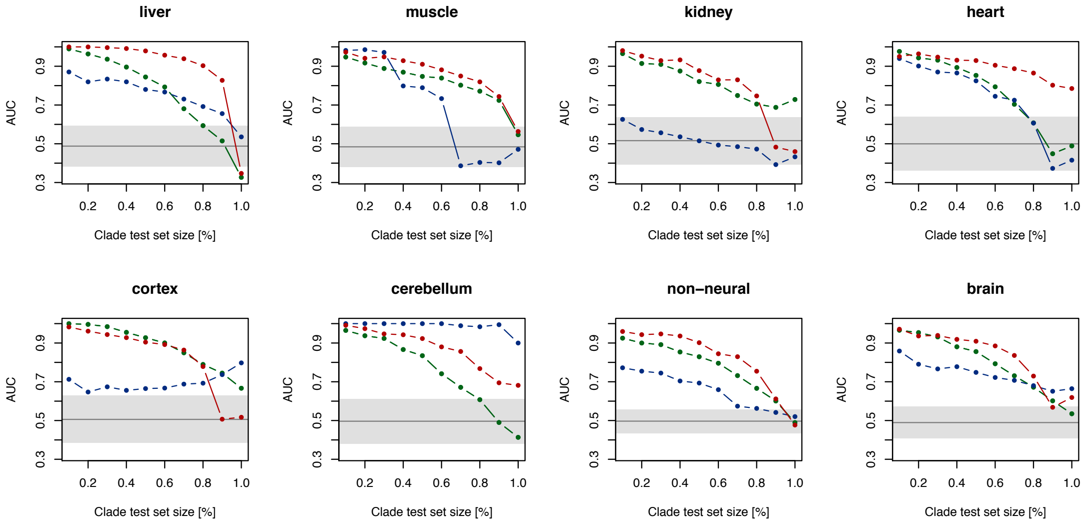

Figure S9. AUC of predictive models presented in Figure S8.

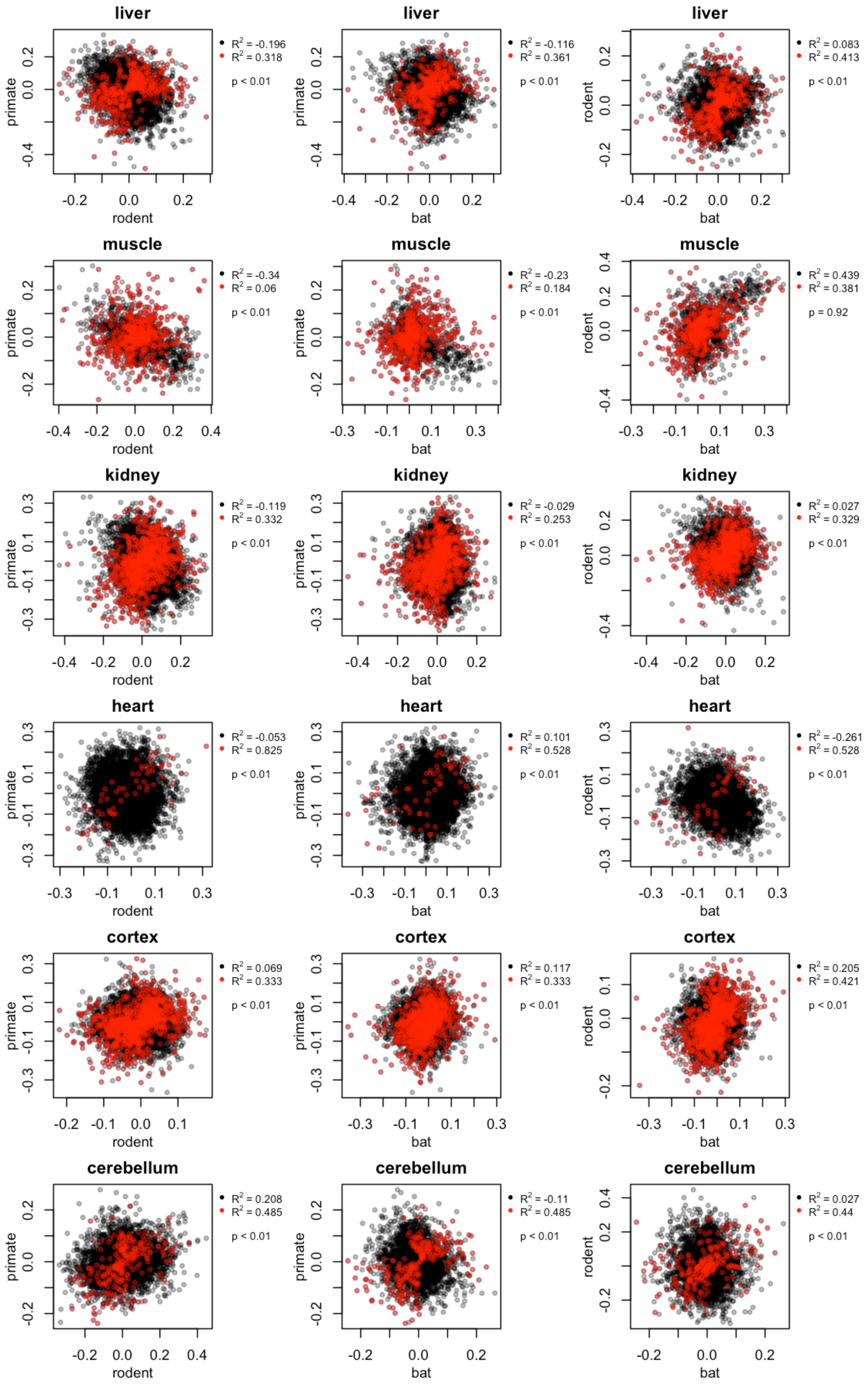

Figure S10. Correlation of the concentration level change in long-living species of

different clades. Change in the concentration levels shown in the x- and y-axes is calculated as the difference between mean of the concentration levels in the long-living species and all other species in each of the clades. Red dots represent predictors of long MLS, black dots – the remaining lipids. Corresponding correlation values for long MLS predictors and the other lipids (R^2), as well as p-value of the correlation of the long MLS predictors, are shown next to each plot.

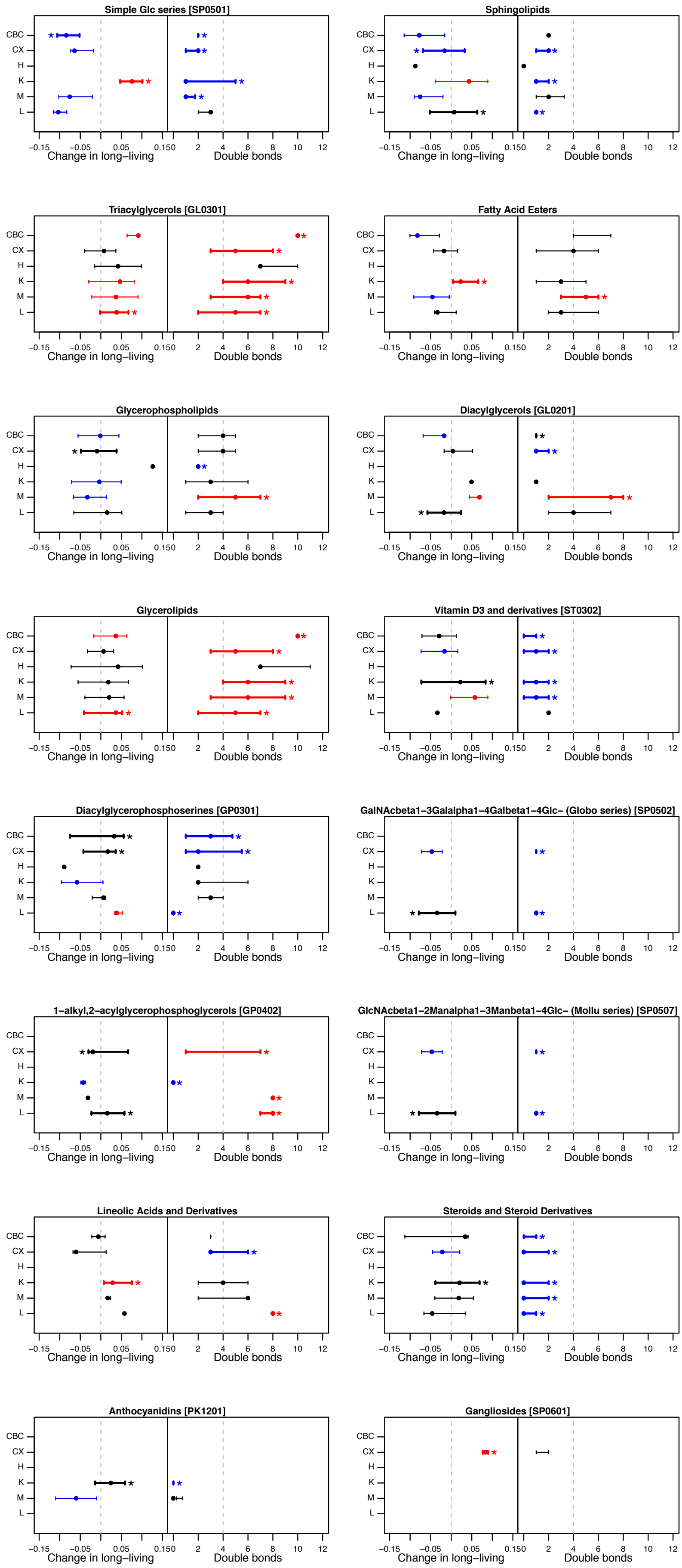
Simple Glc series [SP0501]
Sphingolipids
Triacylglycerols [GL0301]
Fatty Acid Esters
Glycerophospholipids
Diacylglycerols [GL0201]
Glycerolipids
Vitamin D3 and derivatives [ST0302]
Diacylglycerophosphoserines [GP0301]
GalNAcbeta1−3Galalpha1−4Galbeta1−4Glc− (Globo series) [SP0502]
1−alkyl,2−acylglycerophosphoglycerols [GP0402]
GlcNAcbeta1−2Manalpha1−3Manbeta1−4Glc− (Mollu series) [SP0507]
Lineolic Acids and Derivatives
Steroids and Steroid Derivatives
Anthocyanidins [PK1201]
Gangliosides [SP0601]
CBC
CX
H
K
M
L
−0.15 −0.05 0.05 0.15
0 2 4 6 8 10 12
Change in long−living
Double bonds

Figure S11. Concentration level change in long-living species and double bond distribution of long MLS predictors belonging to different lipid sub-classes. Shown are lipid sub-classes enriched in at least one tissue. Change in the concentration levels is calculated as the difference between mean of the concentration levels in the long-living species and all other species in each of the clades. Left panels indicate median and 0.25 to 0.75 inter-quintile regions of the concentration level change for long MLS predictors belonging to a given sub-class of in different tissues. Significant shift of the concentration level change towards lower and higher values in long-living species is indicated by blue and red colors respectively. Significant enrichment of the sub-class compounds among long MLS predictors is indicated by thick line and an asterisk next to the tissue symbol. Vertical dashed line indicates 0. Right panels indicate the double bond number median and 0.25 to 0.75 inter-quintile regions of the corresponding lipids in the left panel. Significantly higher or lower number of double bonds in a given group of lipids is indicated by red and blue color respectively as well as thick line and an asterisk. Vertical dashed line indicates the median of concentration level change and number of double bonds in all long-lifespan predictors.

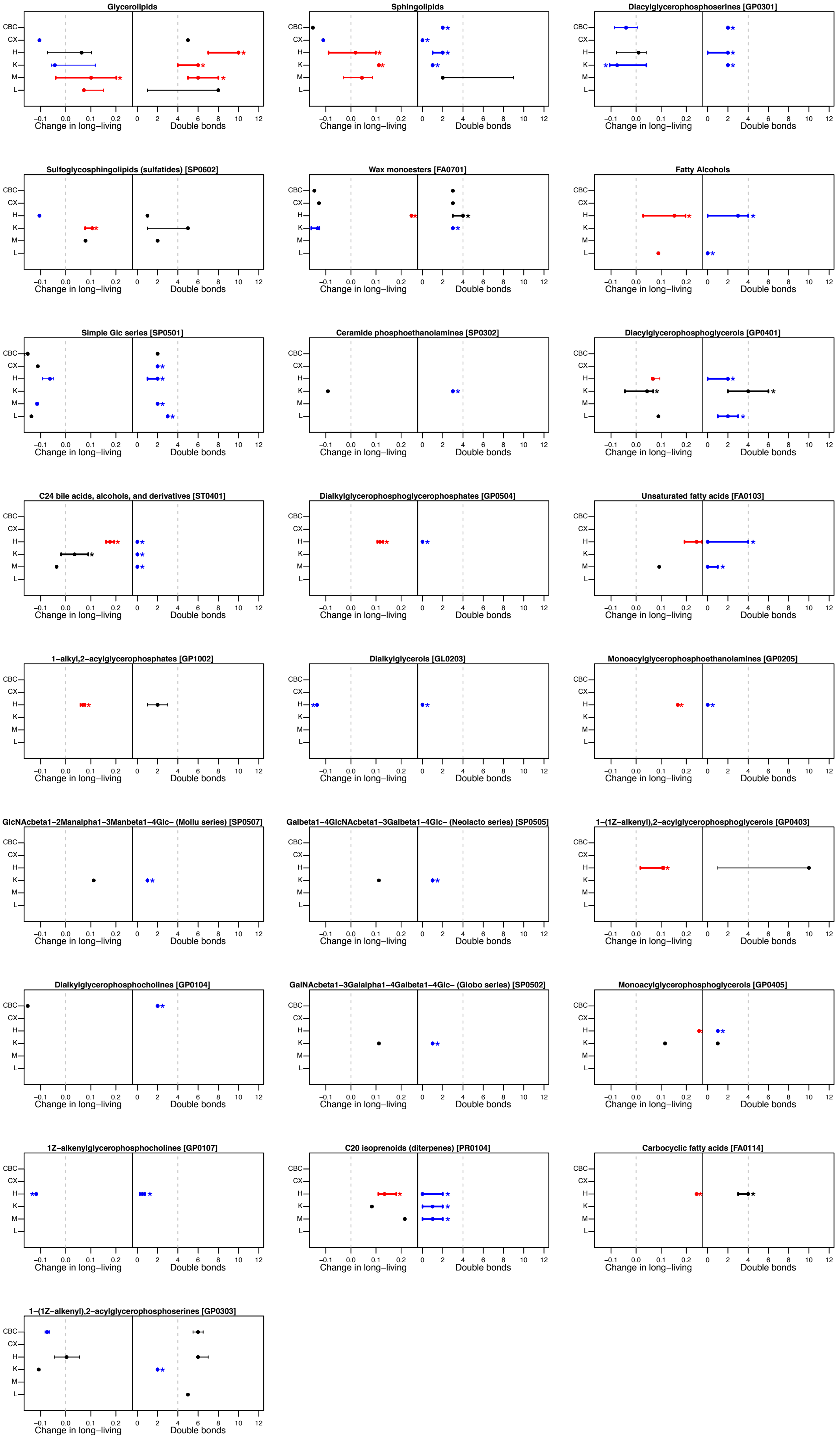
Glycerolipids
Sphingolipids
Diacylglycerophosphoserines [GP0301]
Sulfoglycosphingolipids (sulfatides) [SP0602]
Wax monoesters [FA0701]
Fatty Alcohols
Simple Glc series [SP0501]
Ceramide phosphoethanolamines [SP0302]
Diacylglycerophosphoglycerols [GP0401]
C24 bile acids, alcohols, and derivatives [ST0401]
Dialkylglycerophosphoglycerophosphates [GP0504]
Unsaturated fatty acids [FA0103]
1–alkyl,2–acylglycerophosphates [GP1002]
Dialkylglycerols [GL0203]
Monoacylglycerophosphoethanolamines [GP0205]
GlcNAcbeta1–2Manalpha1–3Manbeta1–4Glc– (Mollu series) [SP0507]
Galbeta1–4GlcNAcbeta1–3Galbeta1–4Glc– (Neolacto series) [SP0505]
1–(1Z–alkenyl),2–acylglycerophosphoglycerols [GP0403]
Dialkylglycerophosphocholines [GP0104]
GalNAcbeta1–3Galalpha1–4Galbeta1–4Glc– (Globo series) [SP0502]
Monoacylglycerophosphoglycerols [GP0405]
1Z–alkenylglycerophosphocholines [GP0107]
C20 isoprenoids (diterpenes) [PR0104]
Carbocyclic fatty acids [FA0114]
1–(1Z–alkenyl),2–acylglycerophosphoserines [GP0303]
CBC
CX
H
K
M
L
Change in long–living
Double bonds

Figure S12. Concentration level change in long-living species and double bond distribution of long MLS predictors in the full lipidome dataset before removal of the peaks potentially related to confounders. The same analysis pipeline was repeated on the full lipidome dataset from which no peaks potentially related to confounding factors were removed. Figure annotation is the same as in Figure S11.

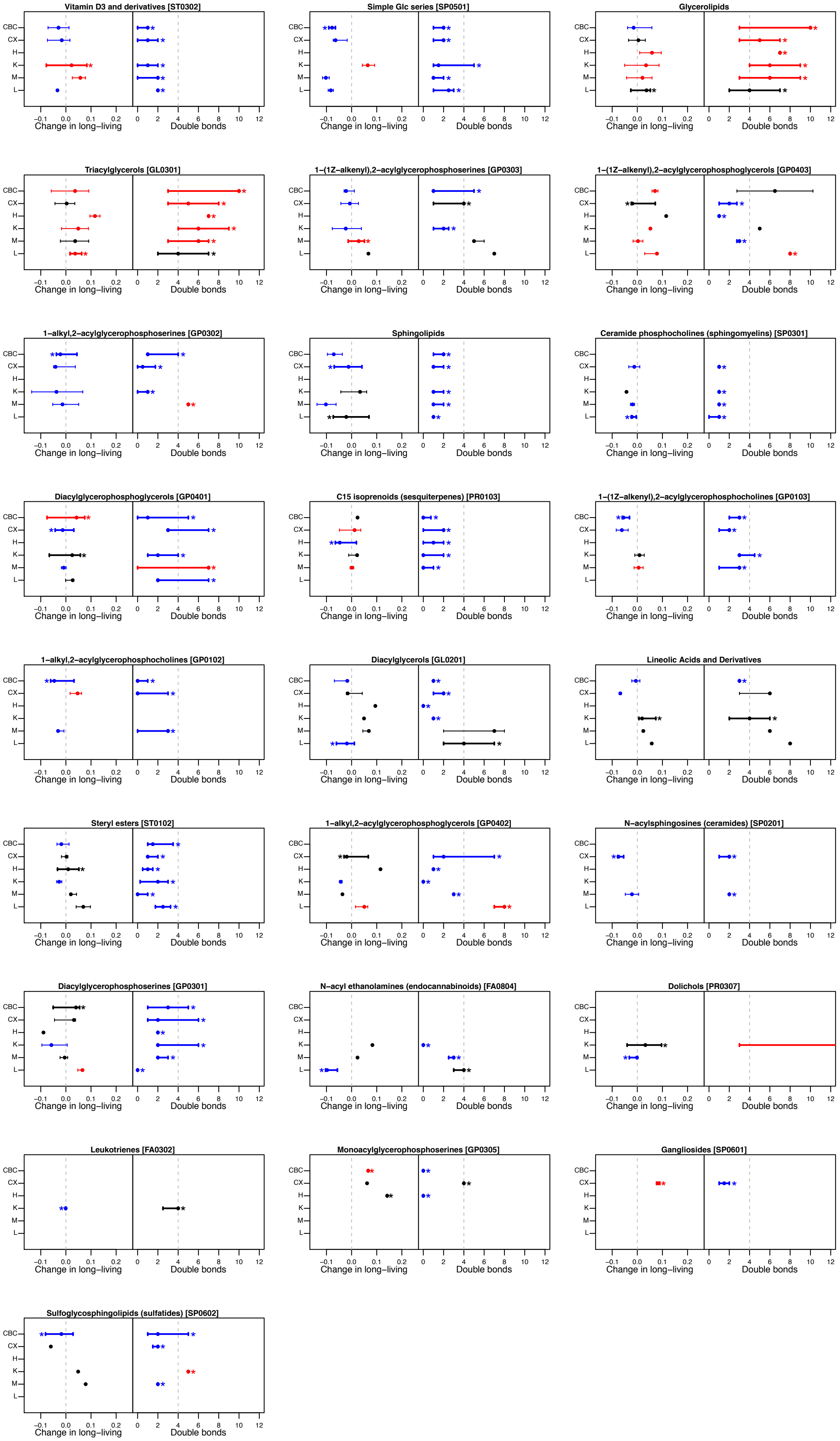
Vitamin D3 and derivatives [ST0302]
Simple Glc series [SP0501]
Glycerolipids
Triacylglycerols [GL0301]
1–(1Z–alkenyl),2–acylglycerophosphoserines [GP0303]
1–(1Z–alkenyl),2–acylglycerophosphoglycerols [GP0403]
1–alkyl,2–acylglycerophosphoserines [GP0302]
Sphingolipids
Ceramide phosphocholines (sphingomyelins) [SP0301]
Diacylglycerophosphoglycerols [GP0401]
C15 isoprenoids (sesquiterpenes) [PR0103]
1–(1Z–alkenyl),2–acylglycerophosphocholines [GP0103]
1–alkyl,2–acylglycerophosphocholines [GP0102]
Diacylglycerols [GL0201]
Lineolic Acids and Derivatives
Steryl esters [ST0102]
1–alkyl,2–acylglycerophosphoglycerols [GP0402]
N–acylsphingosines (ceramides) [SP0201]
Diacylglycerophosphoserines [GP0301]
N–acyl ethanolamines (endocannabinoids) [FA0804]
Dolichols [PR0307]
Leukotrienes [FA0302]
Monoacylglycerophosphoserines [GP0305]
Gangliosides [SP0601]
Sulfoglycosphingolipids (sulfatides) [SP0602]
CBC
CX
H
K
M
L
−0.1 0.0 0.1 0.2
Change in long–living
0 2 4 6 8 10 12
Double bonds

Figure S13. Concentration level change in long-living species and double bond distribution of long MLS predictors in the partial lipidome dataset after removal of the peaks potentially related to all confounders except the body size. Figure annotation is the same as in Figure S11.

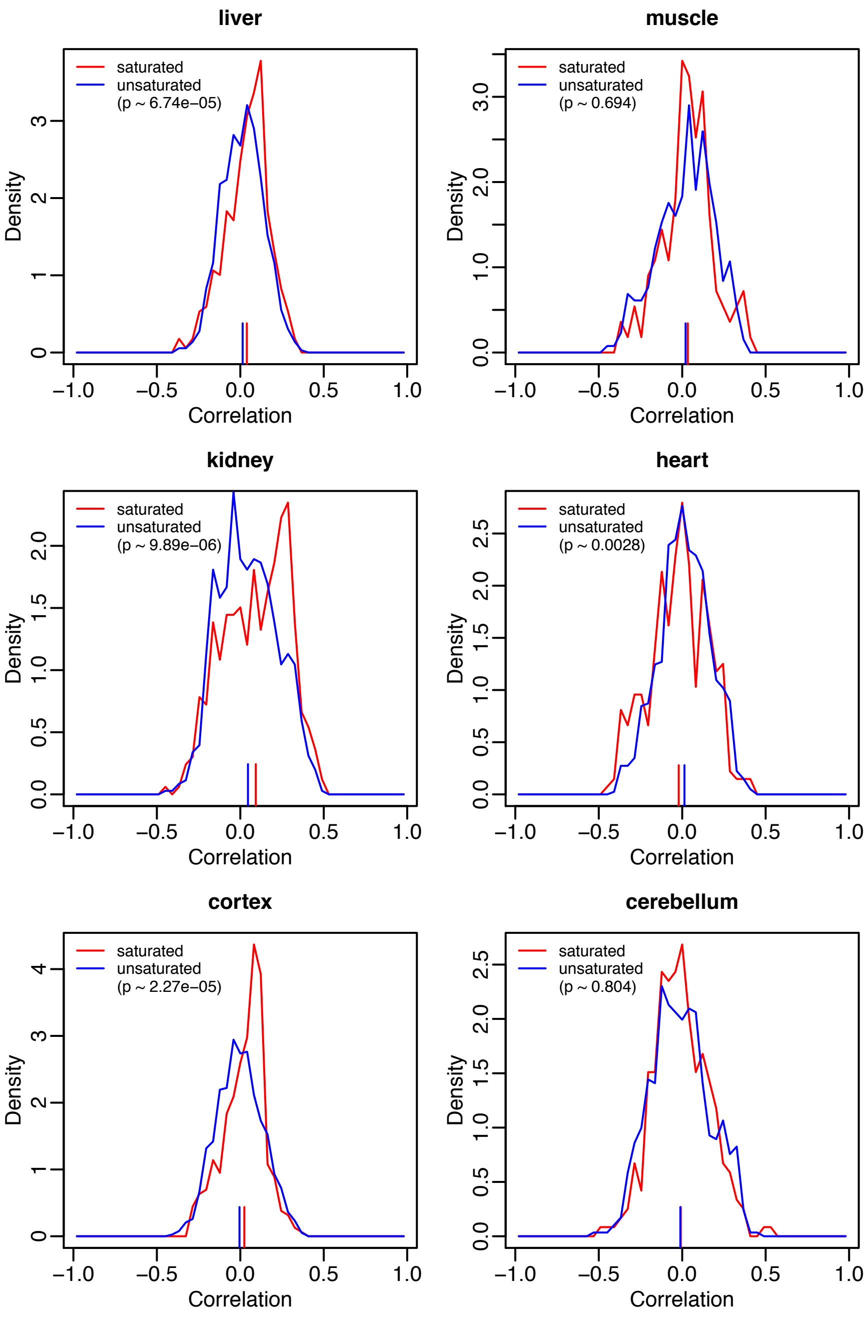

Figure S14. Correlation of the lipid concentration level and species lifespan of the saturated and unsaturated lipids. Correlation distribution is shown in red and blue for the saturated and unsaturated lipids respectively. Mean values of the distributions are marked with vertical lines at the bottom of the plot with respective colors. P-values of the difference between the two distributions are indicated in the legends.

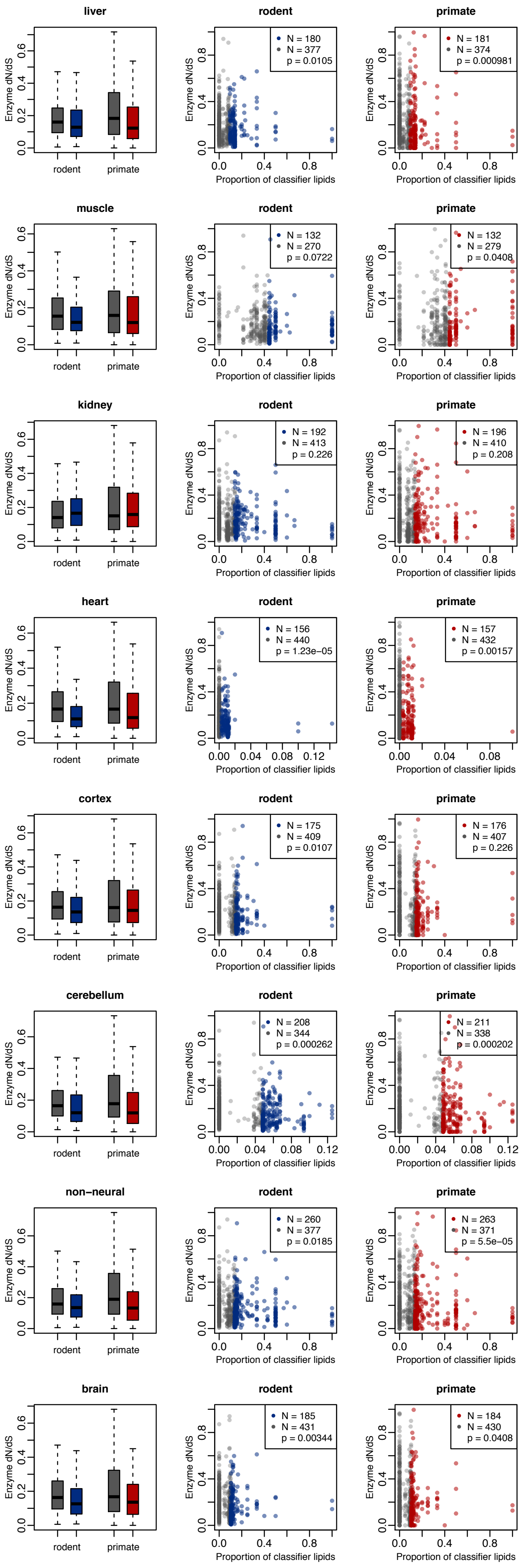
liver
rodent
primate
N = 180
N = 377
p = 0.0105
N = 181
N = 374
p = 0.000981
muscle
N = 132
N = 270
p = 0.0722
N = 132
N = 279
p = 0.0408
kidney
N = 192
N = 413
p = 0.226
N = 196
N = 410
p = 0.208
heart
N = 156
N = 440
p = 1.23e−05
N = 157
N = 432
p = 0.00157
cortex
N = 175
N = 409
p = 0.0107
N = 176
N = 407
p = 0.226
cerebellum
N = 208
N = 344
p = 0.000262
N = 211
N = 338
p = 0.000202
non−neural
N = 260
N = 377
p = 0.0185
N = 263
N = 371
p = 5.5e−05
brain
N = 185
N = 431
p = 0.00344
N = 184
N = 430
p = 0.0408
Enzyme dN/dS
Proportion of classifier lipids

Figure S15. Distribution of the dN/dS values in the long-living species of the enzymes linked to the long MLS predictors, and the enzymes linked to other lipid compounds detected in our data. Scatterplots of the dN/dS ratios (y-axis) and the proportion of the number of the long MLS predictors to the number of all lipids this enzyme is linked to (x-axis) are shown in the second and third column for rodents and primates, respectively. Coloring of the symbols distinguishes lifespan-related enzymes (blue or red) and other lipid enzymes (gray). We defined lifespan-related enzymes as enzymes with top 30% (top 25% in heart and top 35% in non-neural tissues) proportions of the long MLS predictors in a given tissue.

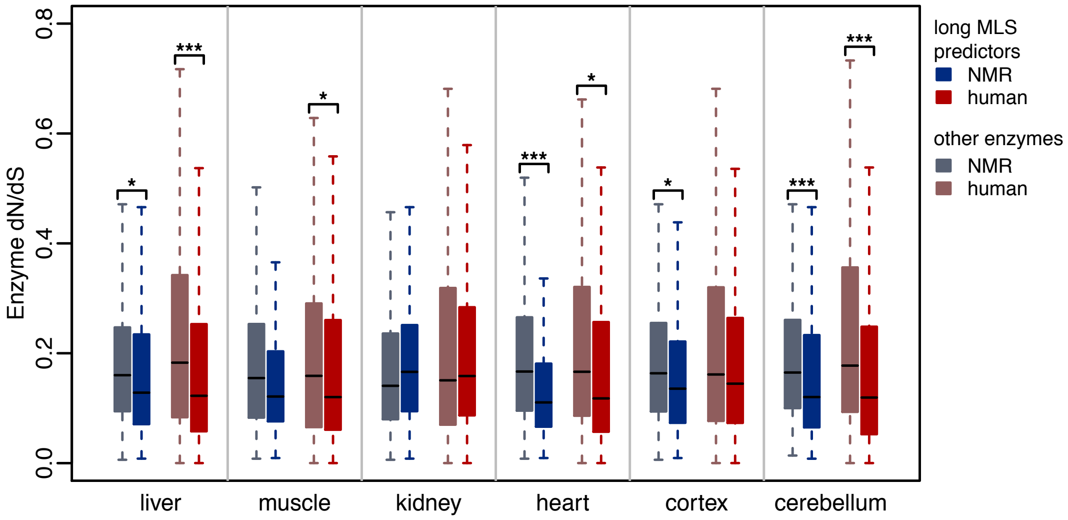

Figure S16. As in Figure 3: Distributions of the dN/dS values of the enzymes linked to the long MLS predictors (darker colors), and the enzymes linked to other lipids detected in our dataset (lighter colors), in the long-living species. Asterisks indicate p-value range * $p < 0.05$, ** $p < 0.01$, *** $p < 0.001$.

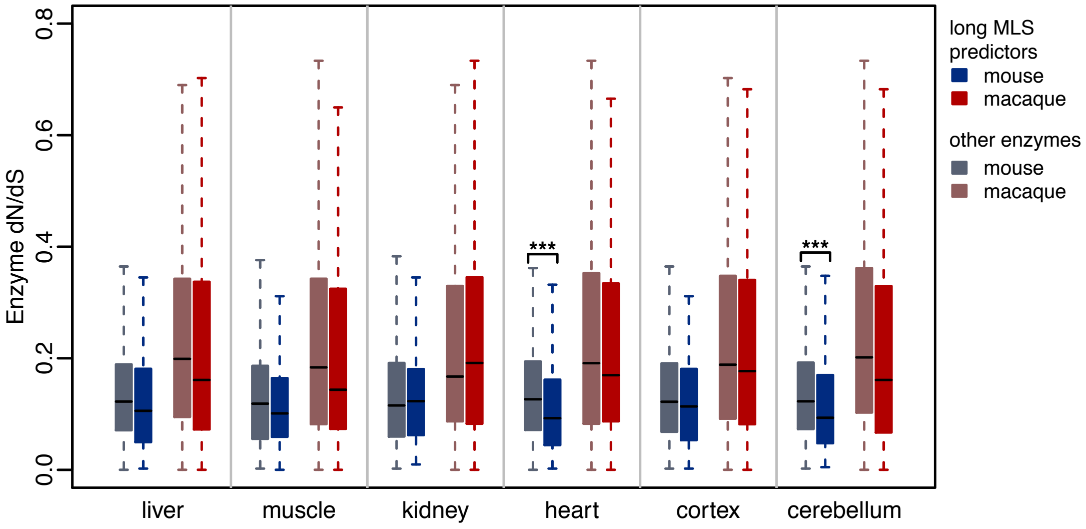

Figure S17. As in Figure 3: distribution of the dN/dS values of the enzymes linked to the long MLS predictors (darker colors), and the enzymes linked to other lipids detected in our dataset (lighter colors) in the short-living species. Asterisks indicate p-value range * p < 0.05, ** p < 0.01, *** p < 0.001.

List of Supplemental Tables

Table S1. Summary table listing tissue samples used in our study.

Table S2. Detailed sample information. This table is provided as separate file.

Table S3. All lipid measurements. Last columns in all six tables list database identifiers for the compounds that could be recognized as known lipids based on their mass to charge ratio. This table is provided as a separate file (also available under http://www.picb.ac.cn/~bozekkasia/lipidome_lifespan/) contains multiple worksheets.

Table S4. P-values of relationships between lipid compounds' concentrations and the values of confounders. This table is provided as a separate file (also available under http://www.picb.ac.cn/~bozekkasia/lipidome_lifespan/) contains multiple worksheets.

Table S5. Agreement between prediction methods: logistic regression, linear regression and SVM.

Table S6. List of tissue samples used to test lipid concentration change with age. This table is provided as separate file.

Table S7. Lipid measurements used to test lipid concentration change with age. Columns *intercept, coef1, coef2, coef3*, list coefficients of the polynomial function predicting lipid concentration level: $f(x) = intercept + coef1 * x + coef2 * x^2 + coef3 * x^3$, where x is the individual age. Where no significant fit of a polynomial could be found *coef1, coef2, coef3* are equal 0 and *intercept* represents the mean value of concentration in all individuals. Matching peaks in the heart and cortex datasets, matched based on their mass to charge ratio and retention time, are listed in the last column. This table is provided as separate file (also available under http://www.picb.ac.cn/~bozekkasia/lipidome_lifespan/) contains multiple worksheets.

Table S8. Overlap of PMD-related peaks and MLS predictors.

Table S9. Coefficients of all peaks in the models predicting long lifespan in each tissue. This table is provided as separate file, contains multiple worksheets.

Table S10. Overlap of lipid compounds selected as predictors of long lifespan among tissues.

Table S11. Lipid classes, sub-classes and pathways significantly enriched among long MLS predictors. Enrichment $p < 0.05$ is indicated in bold, significant shift in concentration levels towards higher or lower values in the long living species is indicated with red and blue color, respectively. This table is provided as separate file, contains multiple worksheets.

Table S12. Relationship of lipid concentration level change in the long-living species and the number of double bonds. The relationship is tested for all lipid

subclasses present in three datasets: without lipids potentially related to confounding effects (‘confounders removed’), without lipids potentially related to all confounding effects except body size (‘partial dataset’), and full lipid dataset without removing lipids potentially related to any confounding effects (‘full dataset’), shown in the following worksheets of the table. Change in concentration level is calculated as the difference in mean value in the long-living and in other species, significance was assessed through permutations. To indicate shift towards lower values is p-values are displayed as negative and higher with positive values. Significant shifts ($p <= 0.01$) in both double bond number and concentration change are marked in orange for both positive, in blue for both negative, and gray for changes in opposite directions. This table is provided as separate file, contains multiple worksheets.

Table S13. Concentration level change of fatty acids in the long-living species.

Table S14. List of enzymes linked to lipid predictors of long MLS. For each enzyme, the proportion of lipid predictors of long MLS to the number of all lipids that the enzyme is linked to, as well as the quintile of this proportion in all enzymes, are listed. The dN/dS in the long- and short-living primate and rodent species are listed together with the proportion of the dN/dS in long- to short-living species. Thick line indicates the cutoff for the lifespan-related enzymes (above 0.75 quintile in heart, 0.65 in combined non-neural tissues, and 0.7 in remaining tissues) with the proportion of the dN/dS in long- to short-living species < 1 in both primates and rodents. These enzymes were considered for further functional analysis. This table is provided as separate file, contains multiple worksheets.

Table S15. Overlap of enzymes listed in Table S12 as lifespan-related and showing an excess of evolutionary constraint in the long-living species compared to short-living ones across tissues. This table is provided as separate file, contains multiple worksheets.

Table S16. GO terms enriched in enzymes classified as lifespan-related and showing an excess of evolutionary constraint in the long-living species compared to short-living ones. This table is provided as separate file, contains multiple worksheets.

Table S17. dN/dS ratios of the lipid enzymes and their orthologs. This table is provided as separate file, contains multiple worksheets.

Table S18. Adducts used in the first step of annotation.

Table S19. Adducts used in the second step of annotation.

Table S20. Values of the confounder parameters of all species used in this study.

Supplemental Tables

Table S1. Summary table listing tissue samples used in our study. Colors of the MLS columns correspond to the species colors in Figure 1.

group	species	MLS	individuals						species total	clade total
			liver	muscle	cortex	cerebellum	kidney	heart		
bat	greater short-nosed fruit bat	10	5	5	5	5	5	5	30	
	great roundleaf bat	13	5	5	5	5	5	5	30	
	common bent-winged bat	22	5	5	5	5	4	5	29	
	chinese horseshoe bat	30	5	5	5	5	5	5	30	
	ricketts big-footed bat	33	5	5	5	5	5	4	29	148
rodent	fischer rat	4	1	0	1	0	1	1	4	
	norway rat	4	1	0	1	0	1	1	4	
	sprague-dawley rat	4	1	0	1	0	1	1	4	
	mouse	4	10	10	10	10	10	10	60	
	hamster	4	3	0	3	0	3	3	12	
	deer mouse	8	2	0	2	0	2	2	8	
	muskrat	10	2	0	2	0	2	2	8	
	red squirrel	10	3	0	2	0	3	3	11	
	guinea pig	10	2	0	2	0	2	1	7	
	highveld mole-rat	11	4	4	4	0	0	0	12	
	natal mole-rat	11	4	4	4	0	0	0	12	
	cape mole-rat	11.2	4	3	4	0	0	0	11	
	damaraland mole-rat	12	4	4	4	0	0	0	12	
	mashona mole-rat	12	4	4	4	0	0	0	12	
	woodchuck	14	2	0	3	0	3	3	11	
	capybara	15	3	0	4	0	2	4	13	
	paca	16	1	0	1	0	1	1	4	
	chinchilla	17	3	0	3	0	3	3	12	
	fox squirrel	18	2	0	2	0	2	2	8	
	grey squirrel	24	3	0	2	0	3	3	11	
	beaver	24	3	0	3	0	3	3	12	
	naked mole-rat	31	10	3	9	3	6	7	38	286
primate	mouse lemur	15	0	20	0	0	0	0	20	
	callithrix jacchus	16	4	5	5	3	5	0	22	
	macaca mulatta	35	14	11	8	11	8	10	62	
	macaca fascicularis	37	4	4	0	0	4	0	12	

	cebus apella	45	0	1	1	0	0	0	2	
	cebus capucinus	55	1	1	1	1	1	1	6	
	chimpanzee	60	11	18	8	12	5	0	54	
	human	100	10	10	9	10	7	11	57	235
		tissue total	141	127	128	75	102	96	**669**	

Table S5. Agreement between prediction methods: logistic regression (log.), linear regression (lin.), and SVM. Agreement was assessed as the number of overlapping features (ov.) selected by different models in every tissue, in the clades separately, and all clades combined. Numbers of selected and overlapping features, as well as the p-value of the overlap, are listed.

		**model features**				**model features**				**model features**			
		**log.**	**lin.**	**ov.**	**p**	**log.**	**SVM**	**ov.**	**p**	**lin.**	**SVM**	**ov.**	**p**
**liver**	**bat**	50	161	30	<0.01	50	764	45	<0.01	161	764	94	<0.01
	**rodent**	311	182	67	<0.01	311	2132	292	<0.01	182	2132	131	<0.01
	**primate**	859	220	145	<0.01	859	1296	612	<0.01	220	1296	142	<0.01
	**all**	495	388	163	<0.01	495	612	328	<0.01	388	612	162	<0.01
**muscle**	**bat**	26	31	7	<0.01	26	233	21	<0.01	31	233	19	<0.01
	**rodent**	137	152	51	<0.01	137	44	32	<0.01	152	44	18	<0.01
	**primate**	183	259	71	<0.01	183	380	131	<0.01	259	380	97	<0.01
	**all**	654	323	205	<0.01	654	1745	644	<0.01	323	1745	300	<0.01
**kidney**	**bat**	16	177	12	<0.01	16	12	2	<0.01	177	12	7	<0.01
	**rodent**	41	98	6	<0.01	41	3264	38	<0.01	98	3264	79	<0.01
	**primate**	321	215	81	<0.01	321	1239	257	<0.01	215	1239	118	<0.01
	**all**	1041	116	73	<0.01	1041	2964	907	<0.01	116	2964	88	<0.01
**heart**	**bat**	61	164	32	<0.01	61	866	47	<0.01	164	866	80	<0.01
	**rodent**	828	286	106	<0.01	828	2954	673	<0.01	286	2954	191	<0.01
	**primate**	1216	234	234	<0.01	1216	4781	1107	<0.01	234	4781	223	<0.01
	**all**	60	372	26	<0.01	60	779	52	<0.01	372	779	148	<0.01
**cortex**	**bat**	52	157	30	<0.01	52	21	11	<0.01	157	21	10	<0.01
	**rodent**	836	306	173	<0.01	836	106	101	<0.01	306	106	29	<0.01
	**primate**	829	95	56	<0.01	829	5111	817	<0.01	95	5111	88	<0.01
	**all**	1077	371	229	<0.01	1077	49	49	<0.01	371	49	25	<0.01
**cerebellum**	**bat**	71	177	33	<0.01	71	199	44	<0.01	177	199	47	<0.01
	**rodent**	248	286	248	<0.01	248	199	61	<0.01	286	199	66	<0.01
	**primate**	857	216	138	<0.01	857	1039	513	<0.01	216	1039	111	<0.01
	**all**	261	141	60	<0.01	261	559	178	<0.01	141	559	63	<0.01

Table S8. Overlap of PMD-related peaks and MLS predictors. PMD effect was previously assessed by Bozek et al. (Neuron, 2015) either in macaque tissues or based on PMD variation among human, chimpanzee and macaque tissues. For liver and heart that were not included in the PMD study of Bozek et al. we tested the overlap with peaks showing PMD effect in any tissue.

	**matched peaks**	**MLD predictors**	**PMD-related (macaques)**	**overlap**	**p-value**	**PMD-related (all primates)**	**overlap**	**p-value**
liver	45	3	6	1	0.37	8	0	1
muscle	8	4	0	0	1	3	2	0.4
kidney	22	1	1	0	1	5	0	1
heart	37	0	5	0	1	13	0	1
cortex	34	1	5	0	1	13	0	1
cerebellum	60	5	9	0	1	20	2	0.52

Table S10. Overlap of lipid compounds selected as predictors of long lifespan among tissues. Table lists the number of overlapping lipids, with lipid predictors of long lifespan in each tissue and the overlap p-value shown in brackets. Lipids in each comparison are limited to those that can be matched between each tissue dataset based on their mass to charge ratio and retention time. Significant p-values are colored. Cell format: overlapping lipids (lipids tissue in row / lipids tissue in column / overlap p-value).

	**liver**	**muscle**	**kidney**	**heart**	**cortex**	**cerebellum**
**liver**		14 (26 / 191 / 0.017)	0 (12 / 194 / 1)	0 (7 / 7 / 1)	1 (5 / 163 / 0.476)	0 (3 / 29 / 1)
**muscle**	14 (191 / 26 / 0.017)		28 (170 / 73 / 0.056)	0 (83 / 3 / 1)	4 (36 / 60 / 0.611)	2 (26 / 11 / 0.078)
**kidney**	0 (194 / 12 / 1)	28 (73 / 170 / 0.056)		2 (192 / 6 / 0.149)	8 (51 / 183 / 0.328)	0 (26 / 37 / 1)
**heart**	0 (7 / 7 / 1)	0 (3 / 83 / 1)	2 (6 / 192 / 0.149)		3 (8 / 174 / 0.112)	0 (1 / 41 / 1)
**cortex**	1 (163 / 5 / 0.476)	4 (60 / 36 / 0.611)	8 (183 / 51 / 0.328)	3 (174 / 8 / 0.112)		22 (248 / 76 / 0.001)
**cerebellum**	0 (29 / 3 / 1)	2 (11 / 26 / 0.078)	0 (37 / 26 / 1)	0 (41 / 1 / 1)	22 (76 / 248 / 0.001)	

Table S13. Concentration level change of fatty acids (FAs) in the long-living species. Change is calculated as the difference in mean value in the long-living and other species, significance was assessed through permutations. Significant changes (p <= 0.01) are marked in orange.

FA	liver		muscle		kidney		heart		cortex		cerebellum	
	change in long-living	p-value	change in long-living	p-value	change in long-living	p-value	change in long-living	p-value	change in long-living	p-value	change in long-living	p-value
16:0	0.010	0.376	0.040	0.217	0.045	0.143	0.054	0.098	0.016	0.262	0.013	0.279
16:1	0.036	0.126	0.027	0.288	0.035	0.195	0.069	0.053	0.011	0.334	0.016	0.241
16:2	0.043	0.095	0.057	0.140	-0.009	0.356	0.113	0.006	0.000	1.000	0.033	0.111
16:3	0.100	0.007	0.000	1.000	0.007	0.491	0.000	1.000	0.052	0.057	0.025	0.165
18:0	0.031	0.152	0.057	0.140	0.082	0.045	0.059	0.083	0.018	0.245	0.019	0.216
18:1	0.035	0.132	0.037	0.232	0.040	0.167	0.100	0.013	0.019	0.234	0.010	0.316
18:2	0.059	0.048	0.092	0.051	0.028	0.253	0.139	0.002	0.069	0.029	0.017	0.228
18:3	0.065	0.036	0.107	0.031	-0.016	0.293	0.148	0.002	0.016	0.271	0.011	0.299
18:4	0.105	0.005	0.000	1.000	0.032	0.224	0.177	0.000	0.000	1.000	-0.015	0.273
20:0	0.033	0.142	0.069	0.102	0.000	1.000	0.039	0.172	0.035	0.115	0.016	0.238
20:1	0.001	0.522	0.043	0.198	0.061	0.084	0.103	0.010	0.013	0.310	-0.001	0.501
20:2	0.004	0.485	0.066	0.112	0.002	0.540	0.131	0.003	0.023	0.200	0.014	0.259
20:3	0.041	0.105	0.060	0.127	-0.021	0.266	0.171	0.000	-0.004	0.421	0.029	0.138
20:4	0.078	0.019	0.093	0.050	-0.010	0.344	0.216	0.000	0.016	0.262	0.029	0.136
20:5	0.062	0.041	0.075	0.082	-0.047	0.126	0.144	0.002	0.035	0.115	0.011	0.304
20:6	0.019	0.247	0.003	0.605	0.034	0.205	0.019	0.308	0.022	0.208	-0.011	0.320
22:0	0.027	0.180	0.044	0.197	0.064	0.077	0.037	0.184	0.022	0.209	-0.013	0.299
22:1	0.021	0.233	0.072	0.094	0.096	0.030	0.092	0.018	0.050	0.063	0.007	0.362
22:2	-0.017	0.301	0.049	0.176	0.014	0.419	0.142	0.002	0.032	0.135	0.021	0.191
22:3	0.004	0.479	0.044	0.198	-0.026	0.237	0.207	0.000	0.021	0.215	0.031	0.125
22:4	0.099	0.007	0.090	0.054	-0.003	0.409	0.241	0.000	0.026	0.166	0.025	0.159
22:5	0.103	0.006	0.085	0.064	-0.054	0.100	0.204	0.000	0.052	0.055	0.023	0.177
22:6	0.044	0.094	0.062	0.121	-0.064	0.072	0.200	0.000	0.011	0.328	0.003	0.430
24:0	0.018	0.259	-0.003	0.293	0.029	0.251	0.010	0.385	0.005	0.425	-0.018	0.242
24:1	0.007	0.435	0.032	0.257	0.055	0.101	0.075	0.040	0.039	0.098	0.010	0.311
24:2	0.010	0.370	0.074	0.086	0.033	0.211	0.145	0.002	0.053	0.055	0.000	1.000
24:4	0.056	0.055	0.082	0.069	-0.034	0.190	0.190	0.000	-0.007	0.391	0.006	0.369
24:5	0.030	0.157	0.000	1.000	-0.048	0.125	0.145	0.002	0.081	0.017	0.001	0.478
24:6	0.048	0.080	0.117	0.027	-0.039	0.166	0.158	0.001	0.070	0.027	0.016	0.243
26:0	0.009	0.406	-0.022	0.167	0.000	1.000	-0.031	0.267	-0.003	0.430	-0.020	0.228
26:1	-0.023	0.262	0.001	0.643	0.062	0.082	0.071	0.049	0.041	0.091	0.027	0.146
26:4	0.079	0.018	0.000	1.000	0.001	0.548	0.000	1.000	0.020	0.223	0.000	1.000
26:6	-0.004	0.414	0.000	1.000	0.020	0.350	0.000	1.000	0.037	0.110	0.053	0.049
20:	0.000	1.000	0.000	1.000	0.096	0.029	0.000	1.000	0.000	1.000	0.000	1.000

24:3	0.000	1.000	0.000	1.000	0.011	0.450	0.000	1.000	0.031	0.139	0.000	1.000
26:	0.000	1.000	0.000	1.000	0.019	0.353	0.000	1.000	0.000	1.000	0.000	1.000
26:2	0.000	1.000	0.000	1.000	0.015	0.397	0.000	1.000	0.061	0.041	0.000	1.000
26:3	0.000	1.000	0.000	1.000	0.035	0.193	0.000	1.000	0.000	1.000	0.000	1.000
26:5	0.000	1.000	0.000	1.000	0.021	0.332	0.000	1.000	0.072	0.025	0.000	1.000
24:2 b	0.000	1.000	0.000	1.000	0.000	1.000	0.000	1.000	0.000	1.000	-0.010	0.347

Table S18. Adducts used in the first step of annotation. For each class of lipids, a database search was performed using the corresponding adducts.

lipid class	positive mode	negative mode
Other Fatty Acyls [FA00]	M+H/M+NH4/M+Na	M-H/M+oAc-H
Fatty Acids and Conjugates [FA01]	M+NH4/M+Na	M-H
Octadecanoids [FA02]	M+NH4/M+Na	M-H
Eicosanoids [FA03]	M+NH4/M+Na	M-H
Docosanoids [FA04]	M+H/M+NH4/M+Na	M-H/M+oAc-H
Fatty alcohols [FA05]	M+NH4/M+Na	M-H
Fatty aldehydes [FA06]	M+NH4/M+Na	M-H
Fatty esters [FA07]	M+NH4/M+Na	M-H
Fatty amides [FA08]	M+H/M+NH4/M+Na	M-H/M+oAc-H
Fatty nitriles [FA09]	M+H/M+NH4/M+Na	M-H/M+oAc-H
Fatty ethers [FA10]	M+H/M+NH4/M+Na	M-H/M+oAc-H
Hydrocarbons [FA11]	M+H/M+NH4/M+Na	M-H/M+oAc-H
Oxygenated hydrocarbons [FA12]	M+H/M+NH4/M+Na	M-H/M+oAc-H
Fatty acyl glycosides [FA13]	M+H/M+NH4/M+Na	M-H/M+oAc-H
Other Glycerolipids [GL00]	M+H/M+NH4/M+Na	M-H/M+oAc-H
Monoradylglycerols [GL01]	M+H/M+NH4/M+Na	M-H/M+oAc-H
Diradylglycerols [GL02]	M+NH4/M+Na	M+oAc-H
Triradylglycerols [GL03]	M+NH4/M+Na	M+oAc-H
Glycosylmonoradylglycerols [GL04]	M+NH4/M+Na	M+oAc-H
Glycosyldiradylglycerols [GL05]	M+H/M+NH4/M+Na	M-H/M+oAc-H
Other Glycerophospholipids [GP00]	M+H/M+NH4/M+Na	M-H/M+oAc-H
Glycerophosphocholines [GP01]	M+H/M+Na	M+HAc-H
Glycerophosphoethanolamines [GP02]	M+H/M+Na	M-H
Glycerophosphoserines [GP03]	M+NH4/M+Na	M-H
Glycerophosphoglycerols [GP04]	M+NH4/M+Na	M-H
Glycerophosphoglycerophosphates [GP05]	M+H/M+NH4/M+Na	M-H/M+oAc-H
Glycerophosphoinositols [GP06]	M+NH4/M+Na	M-H
Glycerophosphoinositol monophosphates	M+H/M+NH4/M+Na	M-H/M+oAc-H
Glycerophosphoinositol bisphosphates [GP08]	M+H/M+NH4/M+Na	M-H/M+oAc-H
Glycerophosphoinositol trisphosphates [GP09]	M+H/M+NH4/M+Na	M-H/M+oAc-H
Glycerophosphates [GP10]	M+NH4/M+Na	M-H
Glyceropyrophosphates [GP11]	M+H/M+NH4/M+Na	M-H/M+oAc-H
Glycerophosphoglycerophosphoglycerols	M+H/M+NH4/M+Na	M-H/M+oAc-H
CDP-Glycerols [GP13]	M+H/M+NH4/M+Na	M-H/M+oAc-H
Glycosylglycerophospholipids [GP14]	M+H/M+NH4/M+Na	M-H/M+oAc-H
Glycerophosphoinositolglycans [GP15]	M+H/M+NH4/M+Na	M-H/M+oAc-H
Glycerophosphonocholines [GP16]	M+H/M+NH4/M+Na	M-H/M+oAc-H
Glycerophosphonoethanolamines [GP17]	M+H/M+NH4/M+Na	M-H/M+oAc-H
Di-glycerol tetraether phospholipids	M+H/M+NH4/M+Na	M-H/M+oAc-H
Glycerol-nonitol tetraether phospholipids	M+H/M+NH4/M+Na	M-H/M+oAc-H
Oxidized glycerophospholipids [GP20]	M+H/M+NH4/M+Na	M-H/M+oAc-H
Other polyketides [PK00]	M+H/M+NH4/M+Na	M-H/M+oAc-H
Linear polyketides [PK01]	M+H/M+NH4/M+Na	M-H/M+oAc-H
Halogenated acetogenins [PK02]	M+H/M+NH4/M+Na	M-H/M+oAc-H
Annonaceae acetogenins [PK03]	M+H/M+NH4/M+Na	M-H/M+oAc-H
Macrolides and lactone polyketides [PK04]	M+H/M+NH4/M+Na	M-H/M+oAc-H
Ansamycins and related polyketides [PK05]	M+H/M+NH4/M+Na	M-H/M+oAc-H
Polyenes [PK06]	M+H/M+NH4/M+Na	M-H/M+oAc-H
Linear tetracyclines [PK07]	M+H/M+NH4/M+Na	M-H/M+oAc-H
Angucyclines [PK08]	M+H/M+NH4/M+Na	M-H/M+oAc-H

Polyether polyketides [PK09]	M+H/M+NH4/M+Na	M-H/M+oAc-H
Aflatoxins and related substances [PK10]	M+H/M+NH4/M+Na	M-H/M+oAc-H
Cytochalasins [PK11]	M+H/M+NH4/M+Na	M-H/M+oAc-H
Flavonoids [PK12]	M+H/M+NH4/M+Na	M-H/M+oAc-H
Aromatic polyketides [PK13]	M+H/M+NH4/M+Na	M-H/M+oAc-H
Non-ribosomal peptide/polyketide hybrids	M+H/M+NH4/M+Na	M-H/M+oAc-H
Isoprenoids [PR01]	M+H/M+NH4/M+Na	M-H/M+oAc-H
Quinones and hydroquinones [PR02]	M+H/M+NH4/M+Na	M-H/M+oAc-H
Polyprenols [PR03]	M+H/M+NH4/M+Na	M-H/M+oAc-H
Hopanoids [PR04]	M+H/M+NH4/M+Na	M-H/M+oAc-H
Acylaminosugars [SL01]	M+H/M+NH4/M+Na	M-H/M+oAc-H
Acylaminosugar glycans [SL02]	M+H/M+NH4/M+Na	M-H/M+oAc-H
Acyltrehaloses [SL03]	M+H/M+NH4/M+Na	M-H/M+oAc-H
Other acyl sugars [SL05]	M+H/M+NH4/M+Na	M-H/M+oAc-H
Other sphingolipids [SP00]	M+H/M+NH4/M+Na	M-H/M+oAc-H
Sphingoid bases [SP01]	M+H	M-H
Ceramides [SP02]	M+H	M-H
Phosphosphingolipids [SP03]	M+H	M-H
Phosphonosphingolipids [SP04]	M+H	M-H
Neutral glycosphingolipids [SP05]	M+H	M-H
Acidic glycosphingolipids [SP06]	M+H	M-H
Basic glycosphingolipids [SP07]	M+H	M-H
Amphoteric glycosphingolipids [SP08]	M+H/M+NH4/M+Na	M-H/M+oAc-H
Sterols [ST01]	M+NH4/M+H/M-H20+H	M-H
Steroids [ST02]	M+NH4/M+H/M-H20+H	M-H
Secosteroids [ST03]	M+H/M+NH4/M+Na	M-H/M+oAc-H
Bile acids and derivatives [ST04]	M+NH4/M+H/M-H20+H	M-H
Steroid conjugates [ST05]	M+H/M+NH4/M+Na	M-H/M+oAc-H
Lipids	M+H/M+NH4/M+Na	M-H/M+oAc-H
Lipis	M+H/M+NH4/M+Na	M-H/M+oAc-H
Prenol Lipids	M+H/M+NH4/M+Na	M-H/M+oAc-H

Table S19. Adducts used in the second step of annotation. For the lipid compounds assigned to peaks in the first step of the annotation, additional hits were searched for using this expanded list of adducts.

positive mode	charge	mass	negative	charge	mass
M+3H	3	1.007276	M-3H	3	-1.007276
M+2H+Na	3	8.33459	M-2H	2	-1.007276
M+H+2Na	3	15.76619	M-H2O-H	1	-19.01839
M+3Na	3	22.989218	M-H	1	-1.007276
M+2H	2	1.007276	M+Na-2H	1	20.974666
M+H+NH4	2	9.52055	M+Cl	1	34.969402
M+H+Na	2	11.998247	M+K-2H	1	36.948606
M+H+K	2	19.985217	M+FA-H	1	44.998201
M+ACN+2H	2	21.52055	M+oAc-H	1	58.00658
M+2Na	2	22.989218	M+Br	1	78.918885
M+2ACN+2H	2	42.033823	M+TFA-H	1	112.985586
M+3ACN+2H	2	62.547097	2M-H	0.5	-1.007276
M+H	1	1.007276	2M+FA-H	0.5	44.998201
M+NH4	1	18.033823	2M+oAc-H	0.5	58.00658
M+Na	1	22.989218	3M-H	0.333333	1.007276
M+CH3OH+H	1	33.033489			

M+K	1	38.963158			
M+ACN+H	1	42.033823			
M+2Na-H	1	44.97116			
M+IsoProp+H	1	61.06534			
M+ACN+Na	1	64.015765			
M+2K+H	1	76.91904			
M+DMSO+H	1	79.02122			
M+2ACN+H	1	83.06037			
M+IsoProp+Na+H	1	84.05511			
M-H20+H	1	-17.00384			
2M+H	0.5	1.007276			
2M+NH4	0.5	18.033823			
2M+Na	0.5	22.989218			
2M+3H2O+2H	0.5	28.02312			
2M+K	0.5	38.963158			
2M+ACN+H	0.5	42.033823			
2M+ACN+Na	0.5	64.015765			

Table S20. Values of the confounder parameters of all species used in this study.

species	BMR [ml O2 / h]	body temperature [C]	diet	body mass [g]	hiberna-tion
Ricketts big-footed bat	NA	NA	fish	25	1
Chinese horseshoe bat	NA	NA	insects	11	1
Common bent-winged bat	94.3	37.7	insects	13.8	1
Great Roundleaf bat	NA	NA	insects	63	1
Greater short-nosed fruit	18.6	36.5	fruits	70	0
human	16000	37	omnivore	70000	0
chimp	9000	37	omnivore	45000	0
cebus capucinus	NA	NA	plants_animals	3010	0
cebus apella	NA	NA	plants_animals	2760	0
macaca fascicularis	NA	37.6	omnivore	4590	0
macaca mulatta	2239	37.3	plants_animals	6450	0
callithrix jacchus	152	36	plants	291	0
mouse lemur	125.84		plants_insects	66.6	1
naked mole-rat	20.5	32.1	plants	32	0
grey squirrel	369.6	38.7	plants	440	0
beaver	18500	NA	plants	NA	0
fox squirrel	NA	NA	plants_animals	800	0
chinchilla	200.2	35.7	plants	426	0
paca	2746.8	37.2	plants	9156	0
capybara	6596.3	37.1	plants	26385	1
woodchuck	662.5	37	plants_insects	2650	1
damaraland mole-rat	78.7	35.2	plants_insects	138	0
mashona mole-rat	58.8	33.3	NA	60	0
cape mole-rat	115.7	36.4	plants_insects	195	0
highveld mole-rat	67.36	NA	NA	73.8	0
natal mole-rat	81.6	NA	NA	102	0
muskrat	642.9	37.4	plants	1004.6	0
red squirrel	NA	NA	plants	333	0
guinea pig	346	39	plants	629	0
deer mouse	36.9	36.6	plants_insects	20.5	0
mouse	56.76	36.9	omnivore	19.3	0
hamster	231.7	39.5	plants_insects	362	1
fischer rat	NA	NA	plants_insects	NA	0
norway rat	257	37.1	plants_insects	283	0
sprague-dawley rat	NA	NA	plants_insects	NA	0
